# At the metal–metabolite interface in *Aspergillus fumigatus*: towards untangling the intersecting roles of zinc and gliotoxin

**DOI:** 10.1099/mic.0.001106

**Published:** 2021-11-05

**Authors:** Aimee M. Traynor, Rebecca A. Owens, Claudia M. Coughlin, Maeve C. Holton, Gary W. Jones, José A. Calera, Sean Doyle

**Affiliations:** ^1^​ Department of Biology, Maynooth University, Maynooth, Co. Kildare, Ireland; ^2^​ Centre for Biomedical Science Research, School of Clinical and Applied Sciences, Leeds Beckett University, Leeds, UK; ^3^​ Instituto de Biología Funcional y Genómica (IBFG-CSIC), Universidad de Salamanca, Salamanca, Spain; ^4^​ Departamento de Microbiología y Genética, Universidad de Salamanca, Salamanca, Spain

**Keywords:** BGC, fungal drug targets, gliotoxin, nutritional immunity, quantitative proteomics, zinc

## Abstract

Cryptic links between apparently unrelated metabolic systems represent potential new drug targets in fungi. Evidence of such a link between zinc and gliotoxin (GT) biosynthesis in *Aspergillus fumigatus* is emerging. Expression of some genes of the GT biosynthetic gene cluster *gli* is influenced by the zinc-dependent transcription activator ZafA, zinc may relieve GT-mediated fungal growth inhibition and, surprisingly, GT biosynthesis is influenced by zinc availability. In *A. fumigatus*, dithiol gliotoxin (DTG), which has zinc-chelating properties, is converted to either GT or *bis*-dethiobis(methylthio)gliotoxin (BmGT) by oxidoreductase GliT and methyltransferase GtmA, respectively. A double deletion mutant lacking both GliT and GtmA was previously observed to be hypersensitive to exogenous GT exposure. Here we show that compared to wild-type exposure, exogenous GT and the zinc chelator *N*,*N*,*N*′,*N*′-tetrakis(2-pyridinylmethyl)−1,2-ethanediamine (TPEN) inhibit *A. fumigatus* Δ*gliT*Δ*gtmA* growth, specifically under zinc-limiting conditions, which can be reversed by zinc addition. While GT biosynthesis is evident in zinc-depleted medium, addition of zinc (1 µM) suppressed GT and activated BmGT production. In addition, secretion of the unferrated siderophore, triacetylfusarinine C (TAFC), was evident by *A. fumigatus* wild-type (at >5 µM zinc) and Δ*gtmA* (at >1 µM zinc) in a low-iron medium. TAFC secretion suggests that differential zinc-sensing between both strains may influence fungal Fe^3+^ requirement. Label-free quantitative proteomic analysis of both strains under equivalent differential zinc conditions revealed protein abundance alterations in accordance with altered metabolomic observations, in addition to increased GliT abundance in Δ*gtmA* at 5 µM zinc, compared to wild-type, supporting a zinc-sensing deficiency in the mutant strain. The relative abundance of a range of oxidoreductase- and secondary metabolism-related enzymes was also evident in a zinc- and strain-dependent manner. Overall, we elaborate new linkages between zinc availability, natural product biosynthesis and oxidative stress homeostasis in *A. fumigatus*.

## Introduction

Gliotoxin (GT) is a non-ribosomal peptide which causes host cell death by a range of mechanisms including intracellular redox activity and direct protein inactivation via thiol interaction and is consequentially referred to as a virulence factor [1]. However, two additional mechanisms by which GT effects a potent and negative influence on the viability of target cells, but which have received limited attention, are now known. The first of these is that upon uptake, GT is converted to reduced or dithiol GT (DTG) by chemical reduction mediated by intracellular reductants such as glutathione (GSH). This leads to depletion of intracellular GSH in animal cells, which itself can result in redox stress, or reactive oxygen species (ROS) [2] production. Secondly, and of equivalent or possibly greater relevance, is that this disulphide cleavage event results in the sudden intracellular presence of a zinc chelator, DTG, which can both chelate intracellular zinc and eject it from cellular zinc-metalloenzymes [3] – potentially disrupting intracellular zinc homeostasis and resulting in disrupted enzyme activity, respectively [4]. This nascent scenario, that it is DTG, rather than GT, which is the actual deleterious form of the natural product, merits serious consideration and may be related to the role of GT and related epipolythiodioxopiperazines in fungi and dithiolopyrrolones (DTPs; e.g. holomycin) in bacteria [5–7].

Several groups have recently established a connection between zinc availability and GT biosynthesis in the opportunistic fungal pathogen *Aspergillus fumigatus*. In addition to demonstrating the zinc chelation potential of DTG, Saleh *et al*. [3] revealed that zinc addition to culture media suppressed GT biosynthesis in *A. fumigatus*. Simultaneously, Vicentefranqueira *et al*. [8] reported that amongst other effects, expression of certain genes of the *gli* biosynthetic cluster that encodes GT biosynthesis, specifically *gliZ* (encoding the Zn2-Cys6-transcription factor that regulates expression of nearly all *gli* genes), *gliA* (encoding a plasma membrane GT exporter), *gliT* (encoding a GT oxidoreductase for GT biosynthesis) and *gtmA* (a SAM-dependent DTG methyltransferase GtmA, termed TmtA in [9]) are regulated by ZafA, which is the zinc-responsive transcription factor that controls the adaptive response to zinc starvation in *A. fumigatus* [8]. Later, the observed increased expression of genes involved in zinc acquisition in *A. fumigatus* Δ*gliP*, deficient in GT biosynthesis, led to the suggestion by Liu *et al*. [10] of a potential role for GT in zinc acquisition. DTG has been shown to induce anoikis of cancer cells in a ROS-dependent manner [11] and to inhibit neutrophil leukotriene A4 hydrolase activity, a zinc-metalloenzyme, possibly by zinc ejection, which impeded formation of the chemoattractant LTB_4_ and consequently impaired neutrophil function [12]. Lastly, Chan *et al*. [7] revealed a functionally equivalent role for dithiol holomycin whereby metallo-β-lactamase activity was similarly attenuated *in vitro*, which infers a role for reduced DTPs in inhibiting bacterial antibiotic resistance.

There is also a potential impact of DTG-mediated zinc chelation on oxidative stress and redox cycling within both *A. fumigatus* and infected host cells. Indeed, evidence that zinc plays an important role in the protection against oxidative stress is available [13, 14]. Additionally, the oxidative stress response in *A. fumigatus* includes the activity of several enzyme classes, including superoxide dismutases (SODs), catalases, and members of the thioredoxin and glutathione systems [15–17]. Zinc is essential for ROS dismutation-related enzyme activity, including several *A. fumigatus* and mammalian SODs [18–20]. Relatedly, Owens *et al*. [21] showed that co-addition of GT and H_2_O_2_ significantly attenuated H_2_O_2_-mediated ROS formation in mycelial cultures when compared to H_2_O_2_ alone. Thus, further investigation into the relationship between this GT, metal ions and ROS metabolism could provide interesting insight into cell protection mechanisms and novel potential antifungal targets.

Doyle *et al*. [22] revealed that extended culturing of *A. fumigatus* Δ*gtmA*, incapable of DTG *bis*-thiomethylation, resulted in significant remodelling of the fungal secondary metabolome. Interestingly, although the precise mechanistic reason for these observations was opaque, the authors noted a time-dependent and significantly increased abundance of the ZrfA zinc transporter in mycelial extracts. This led us to speculate that zinc depletion may have occurred during extended cultures, which inferred a further link between secondary metabolite formation and zinc. Indeed, Dolan *et al*. [23, 24] observed that GtmA-mediated formation of BmGT attenuates GT biosynthesis, and that elevated intracellular GT results from *gtmA* absence. Thus, any increase in intracellular GT, or possibly DTG, levels in *A. fumigatus* Δ*gtmA* is hypothesized to be a contributing factor to this zinc depletion. Combined, all these findings underpin a new paradigm linking GT biosynthesis and zinc availability and/or zinc homeostasis in *A. fumigatus*. Here, we investigate phenotypic and proteomic responses of *A. fumigatus* wild-type, Δ*gliT*Δ*gtmA* and Δ*gtmA* to zinc stress to further explore this phenomenon.

## Methods

### *A. fumigatus* wild-type and mutant strain phenotypic assays in response to GT, TPEN and zinc

*A. fumigatus* wild-type, Δ*gtmA* and Δ*gliT*Δ*gtmA* strains [23, 24] were grown on malt extract agar (MEA) for 5 days at 37 °C. After incubation, conidia were harvested with 0.1 % Tween in Milli-Q water, washed three times, and then stored in Milli-Q water. Conidia were counted using a haemocytometer and stored at 4 °C for future use. For liquid culture, conidia (2.5×10^5^ conidia ml^–1^ final) were inoculated in synthetic dextrose nitrate (SDN) liquid medium [1.9 g l^−1^ YNB-Zn (Formedium, Cat. CYN2401), 20 g l^−1^ d-glucose, 3 g l^−1^ sodium nitrate, 6 µM FeSO_4_, 2 µM CuSO_4_, 2 µM Na_2_MoO_4_, in Milli-Q water] containing GT [0 or 1 µg ml^−1^ (0 and 3.06 µM)] or *N*,*N*,*N*′,*N*′-tetrakis(2-pyridinylmethyl)-1,2-ethanediamine [TPEN; 0, 125, or 250 ng ml^−1^ (0, 0.3 and 0.58 µM]) and ZnSO_4_ (0, 1 or 5 µM), and incubated at 37 °C and 200 r.p.m. for 48 h (triplicate). For solid-state agar, conidia were serially diluted to 10^3^ conidia ml^–1^ and 5 µl was spotted onto Sabouraud Dextrose (SAB) or SDNE (i.e. SDN liquid medium supplemented with 250 µM EDTA) agar plates containing GT [0, 1, 5 or 10 µg ml^−1^ (0, 3.06, 15.3 and 30.6 µM)] or TPEN [0, 0.25, 0.5, 5 or 10 µg ml^−1^ (0, 0.58, 1.2, 11.8, 23.5 µM]) and ZnSO_4_. EDTA is an extracellular zinc chelator that was included in SDNE agar to chelate residual Zn^2+^ ions, thereby enabling clarity of phenotypic observations. TPEN is a well-established intracellular zinc chelator [25]. Plates were incubated at 37 °C and growth-monitored for 24 h (SAB) or 48 h (SDNE) by measuring colony radial growth (mm). All glassware used to prepare and grow *A. fumigatus* cultures with SDNE agar or SDN liquid medium was incubated overnight in 3 mM EDTA and then extensively rinsed with ultrapure Milli-Q water to ensure metal ion removal. Mycelia were harvested using Miracloth and dried at 70 °C prior to weighing. Two-way ANOVA was performed to determine the statistical significance between strains at different concentrations of GT, TPEN and Zn.

### RP-HPLC detection of natural products from *A. fumigatus* culture supernatants

*A. fumigatus* wild-type and ∆*gtmA* were grown in SDN for 48 h at 37 °C and 200 r.p.m. with varying zinc and exogenous GT concentrations (triplicate). Collected supernatants were centrifuged at 3000 ***g*** for 10 min prior to analysis. Supernatants were analysed by RP-HPLC with UV detection (Agilent 1200 system), using a C8 RP-HPLC column (Agilent Zorbax Eclipse XDB-C8 Analytical; 5 µm particle size; 4.6×150 mm) at a flow rate of 1 ml min^−1^. A mobile phase of water and acetonitrile with 0.1 % (v/v) trifluoroacetic acid was used under various gradient conditions. GT (purity: 98 %) and BmGT (purity: 99 %) standards were obtained from Sigma-Aldrich and Enzo Life Sciences, respectively. All data were analysed using built-in GraphPad prism version 9.1.0 functions, as specified.

### TAFC detection and isolation by RP-HPLC and identification by LC-MS

*A. fumigatus* wild-type and ∆*gtmA* were grown in SDN without iron supplementation for 48 h at 37 °C and 200 r.p.m. with varying zinc concentrations (0, 1, 5, 25 µM). Collected supernatants were centrifuged at 3000 ***g*** for 10 min. FeSO_4_ was added to supernatant aliquots to final concentrations of 10 or 100 µM. Unferrated and ferrated samples were analysed by RP-HPLC as above. Fractions were collected between 12.4 and 12.8 min and subsequently dried before resuspension in LC-MS buffer. LC-MS analysis was performed using a Thermo Fisher Q-Exactive mass spectrometer coupled to a Dionex RSLC-nano with a Hypersil GOLD aQ column (100×2.1 mm, 1.9 µm particle size). LC gradients ran from 5 to 95 % B over 10 min, and data were collected using a Top5 method for MS/MS scans.

### Comparative quantitative proteomic analysis of *A. fumigatus* wild-type and mutant strains

*A. fumigatus* wild-type and ∆*gtmA* (triplicate) were cultured in Czapek-Dox (CD) without a zinc supplement and supplemented with zinc (5 or 25 µM) for 6 days at 37 °C with shaking at 200 r.p.m. Mycelia were harvested and snap frozen in liquid N_2_. Mycelial lysates were prepared in lysis buffer [100 mM Tris-HCl, 50 mM NaCl, 20 mM EDTA, 10 % (v/v) glycerol, 1 mM PMSF, 1 µg ml^−1^ Pepstatin A, pH 7.5] with grinding and sonication, and clarified using centrifugation. The resultant protein lysates were precipitated using trichloroacetic acid/acetone and resuspended in 100 mM Tris-HCl, 6 M urea and 2 M thiourea (pH 8.0). After DTT reduction and iodoacetamide-mediated alkylation, sequencing-grade trypsin combined with ProteaseMax surfactant was added [24, 26]. All peptide mixtures were analysed via a Thermo Fisher Q-Exactive mass spectrometer coupled to a Dionex RSLC-nano. LC gradients ran from 4 to 35 % B over 3 h, and data were collected using a Top15 method for MS/MS scans. Comparative proteome abundance and data analysis was performed using MaxQuant software (Version 1.6.5.0), with Andromeda used for database searching and Perseus used to organize the data (Version 1.6.5.0).

## Results

### *A. fumigatus* wild-type and Δ*gliT*Δ*gtmA* show a differential response to GT, TPEN and altered zinc levels in growth media

Comparison of the growth-inhibitory effects of GT and the membrane-permeable zinc ion chelator TPEN was carried out in the defined zinc-limiting growth medium SDN, and complex zinc-replete medium SAB. Growth media were also supplemented with 1 or 5 µM zinc at varying concentrations of GT and TPEN, respectively. Preliminary experiments on solid-state agar showed hypersensitivity of *A. fumigatus* Δ*gliT*Δ*gtmA* to exogenous GT and an increase in sensitivity to TPEN exposure compared to wild-type. Higher concentrations of both were required to elicit a growth inhibitory response on complex nutrient medium SAB compared to SDNE. Zinc supplementation to agar restored mutant growth in the presence of both GT and TPEN (Fig. S1, available in the online version of this article). These observations led to the development of liquid culture SDN assays to measure growth inhibition and relief as a function of mycelial biomass.

**Fig. 1.**
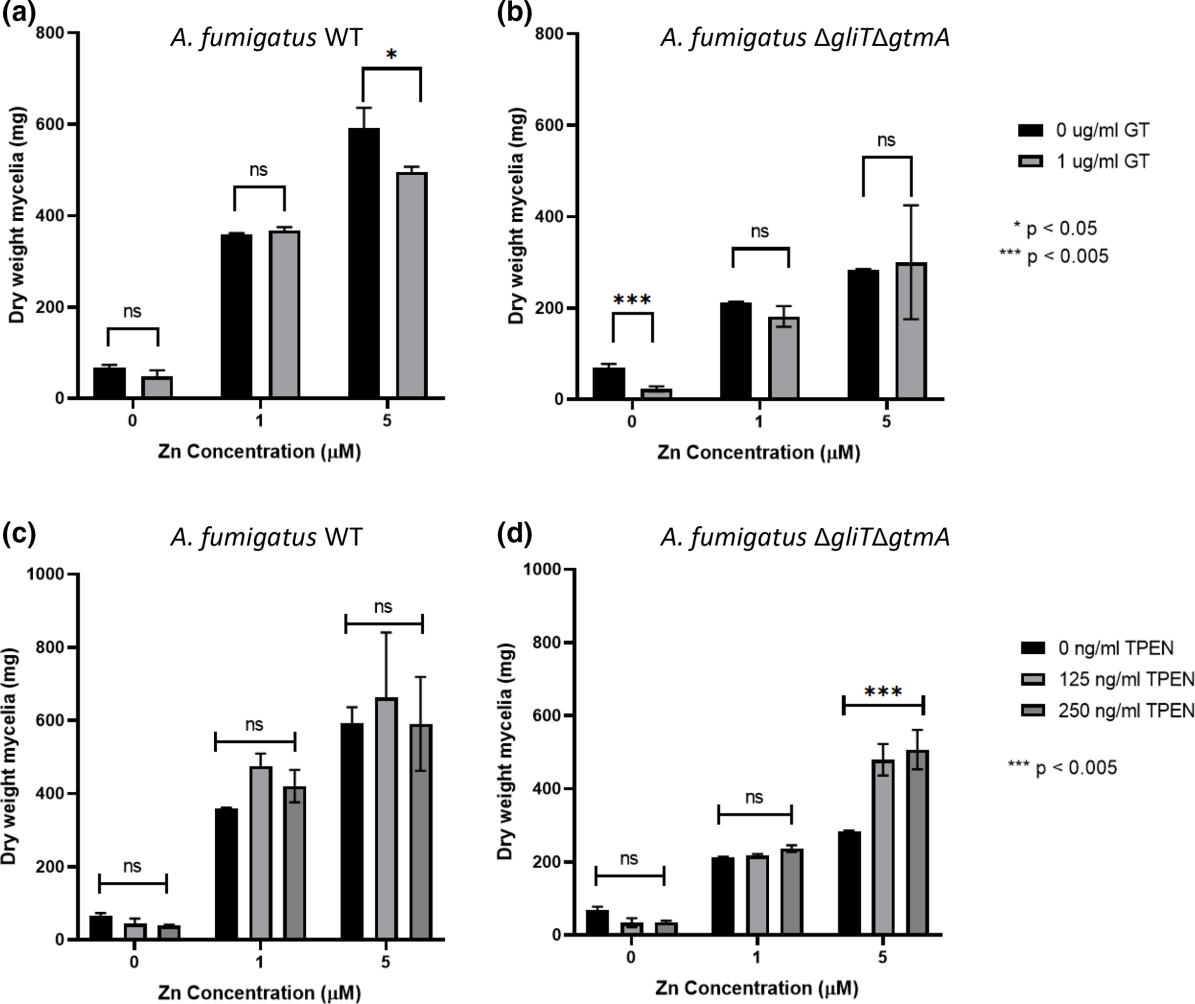
Zinc availability relieves GT- and TPEN-mediated growth inhibition of *A. fumigatus*. Growth of *A. fumigatus* wild-type and Δ*gliT*Δ*gtmA* in SDN in the presence of exogenous GT or TPEN, with or without added Zn. (**a**) Dry mycelial weight (mg) of wild-type in the presence of GT with or without Zn. (**b**) Dry mycelial weight (mg) of double deletion mutant in the presence of GT with or without Zn. (**c**) Dry mycelial weight (mg) of wild-type in the presence of TPEN with or without Zn. (**d**) Dry mycelial weight (mg) of double deletion mutant in the presence of TPEN with or without Zn. *n*=3.

Addition of exogenous GT (1 µg ml^−1^) to SDN significantly reduced the growth of Δ*gliT*Δ*gtmA* by 67 % (*P*=0.001), while wild-type growth was not significantly affected (Fig. 1a, b). Supplementation of SDN with zinc increased the dry weight of both wild-type and double deletion mutant mycelia. The presence of 1 µM zinc, in the absence and presence of GT, relieved the inhibitory effect of GT on *A. fumigatus* Δ*gliT*Δ*gtmA* growth to a non-significant margin (Fig. 1b). The difference between growth in the presence or absence of exogenous GT with 5 µM zinc was also negligible. Since zinc supplementation relieved GT-mediated growth inhibition of Δ*gliT*Δ*gtmA*, this supports the hypothesis [3] that intracellular DTG acts as a zinc-chelator, and possibly interferes with specific zinc-metalloenzyme functionality and inhibits growth.

Incubation of *A. fumigatus* in the presence of the intracellular chelator TPEN [25] allows direct assessment of zinc-chelation effects on growth. With TPEN (125 ng ml^−1^; 0.29 µM), wild-type growth was inhibited by 32 % compared to no TPEN while *A. fumigatus* Δ*gliT*Δ*gtmA* was inhibited by 52 %. Increasing the TPEN concentration to 250 ng ml^−1^ (0.58 µM) did not further inhibit the growth of either wild-type or double mutant in zinc-depleted conditions (Fig. 1c, d). Wild-type growth was less susceptible to TPEN inhibition in media, which suggests a possible combinatorial effect of TPEN- and DTG-mediated zinc-chelation affecting double mutant growth. Growth of both strains was restored to control levels with the supplementation of 1 µM zinc in media, in the presence of both 125 and 250 ng ml^−1^ TPEN. Wild-type growth was also non-significantly different with TPEN at 5 µM zinc supplementation (*P=*0.1751). However, when *A. fumigatus* Δ*gliT*Δ*gtmA* was cultured in the presence of TPEN (125 or 250 ng ml^−1^; 0.29 and 0.58 µM) and 5 µM zinc, growth was significantly increased by 69.8 % (*P<*0.001) and 79.6 % (*P<*0.001), respectively (Fig. 1d). Mycelial growth of the double mutant was generally less than that of the wild-type, but double mutant growth at 5 µM zinc and TPEN (250 ng ml^−1^) was like that of the wild-type under the same conditions. This suggests that adding low levels of metal ion chelators in replete conditions could increase uptake of important metals such as zinc or iron via membrane transporters or secreted metabolites, improving levels of growth. Growth of *A. fumigatus* Δ*gliT*Δ*gtmA* in media containing 5 µM zinc and higher concentrations of TPEN (500 and 1000 ng ml^−1^) did not show a further increase in biomass. In fact, growth was slightly inhibited at 500 ng ml^−1^ and further inhibited at 1000 ng ml^−1^ (Fig. S2).

### Differential *A. fumigatus* wild-type and Δ*gtmA* responses to media zinc supplementation with or without exogenous gliotoxin

**Fig. 2.**
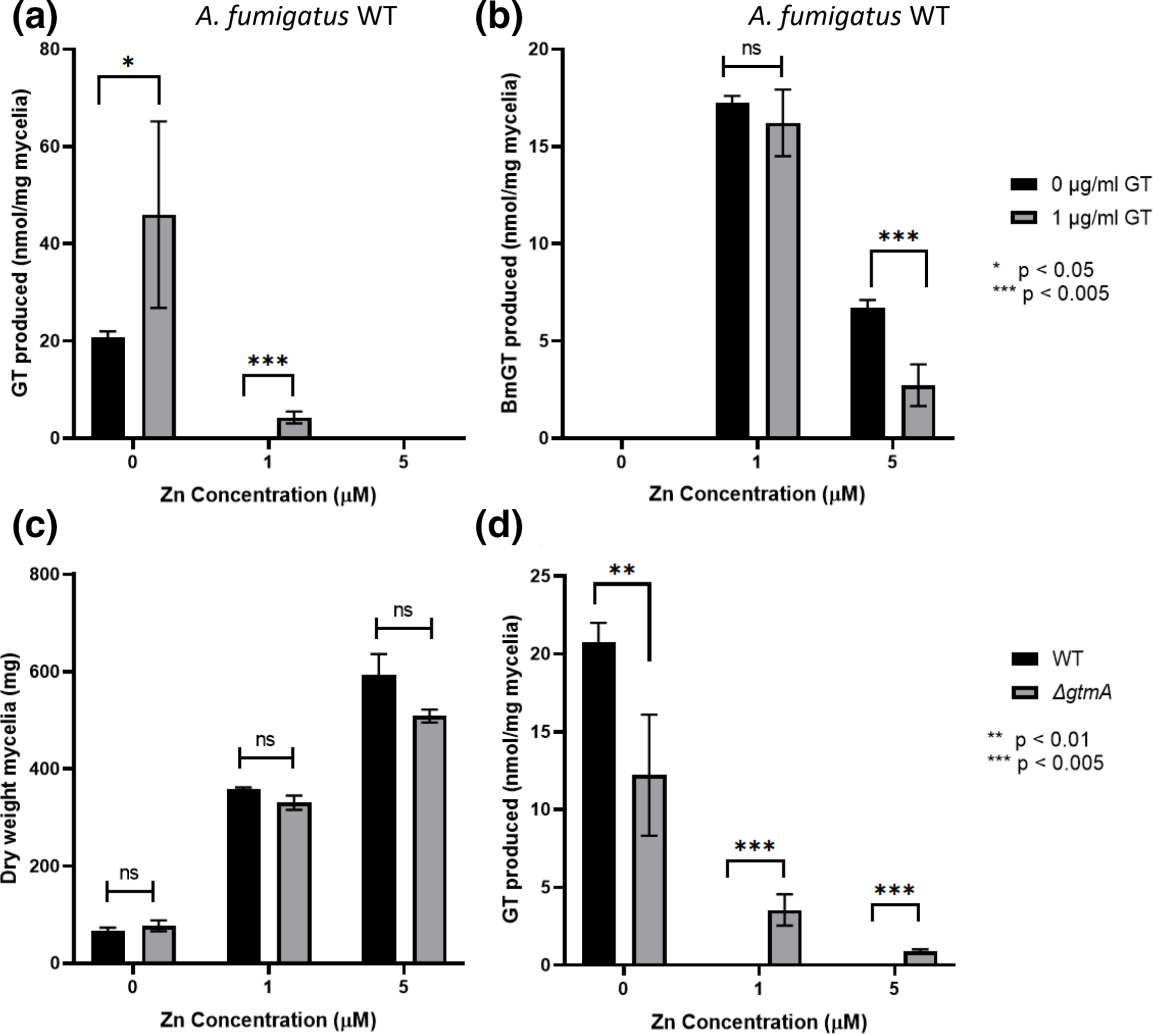
GT biosynthesis influenced by zinc availability and GtmA-mediated formation of BmGT. Detection of GT and BmGT by RP-HPLC from *A. fumigatus* wild-type culture supernatant containing exogenous Zn and GT. (**a**) Production of GT with increasing Zn with or without exogenous GT. (**b**) Production of BmGT with increasing Zn with or without exogenous GT. *n*=3. Differential response of *A. fumigatus* wild-type and Δ*gtmA* to increasing zinc availability. (**c**) Dry mycelial weight (mg) of *A. fumigatus* strains with varying zinc concentrations. (**d**) Detection of GT in supernatant by RP-HPLC. *n*=3.

*A. fumigatus* wild-type produces GT in zinc-limiting SDN and addition of zinc to liquid media decreased the amount of GT produced. Greater than 1 µM zinc in growth media inhibited production and secretion of GT by mycelia. At the same time, BmGT was detectable in the presence of 1 µM zinc (Fig. 2a, b). GtmA negatively regulates GT biosynthesis through BmGT production and secretion [23], and these data suggest a connection between zinc availability and GtmA downregulation of GT biosynthesis. Addition of exogenous GT (1 µg ml^−1^) to growth media was not detectable by RP-HPLC (Fig. S3), yet it significantly increased (*P*=0.043) the concentration of *de novo* GT produced by *A. fumigatus* wild-type. Under zinc-limited growth conditions, GT levels increased from 21 to 46 nmol mg^−1^ mycelia in the presence of exogenous GT (119 % increase). Even at 1 µM zinc, which was shown to inhibit GT biosynthesis, the presence of exogenous GT stimulated production, whereby 4 nmol GT mg^−1^ mycelia was detectable in culture supernatants (*P=*0.003) (Fig. 2a). While exogenous GT positively upregulated its own biosynthesis, it decreased the production of BmGT by wild-type mycelia (Fig. 2b). The concentration of BmGT in the presence of 1 µM zinc decreased from 17 to 16 nmol mg^−1^ mycelia with the addition of exogenous GT (1 µg ml^−1^), and from 8 to 3 nmol mg^−1^ mycelia in the presence of 5 µM zinc (63 % decrease, *P*=0.001) (Fig. 2b). Interestingly, the level of GT produced at no added zinc (0 µM zinc) is comparable to the level of BmGT produced at 1 µM zinc in the absence of exogenous GT. This finding strongly suggests that, in fact, DTG was also synthesized in the presence of a low amount of zinc (e.g. 1 µM) but, unlike in the absence of zinc, all of it was readily converted into BmGT by GtmA rather than into GT by GliT (whose expression is strongly reduced even in the presence of zinc).

*A. fumigatus* Δ*gtmA* showed a somewhat different response to changing zinc concentrations in liquid media. While there was no change in mycelial growth compared to the wild-type with increasing zinc concentrations in media (Fig. 2c), there were significant differences in levels of GT produced. With addition of 0 µM zinc, *A. fumigatus* Δ*gtmA* produced less GT than the wild-type (12 vs. 21 nmol mg^−1^ mycelia respectively, *P*=0.007). This corresponds to a 43 % decrease (Fig. 2d). However, we observed that the GtmA-deficient strain continued to produce low levels of GT even at 5 µM zinc. This suggests that both zinc availability and GT *bis*-methylation are required for complete downregulation of toxin biosynthesis.

**Fig. 3.**
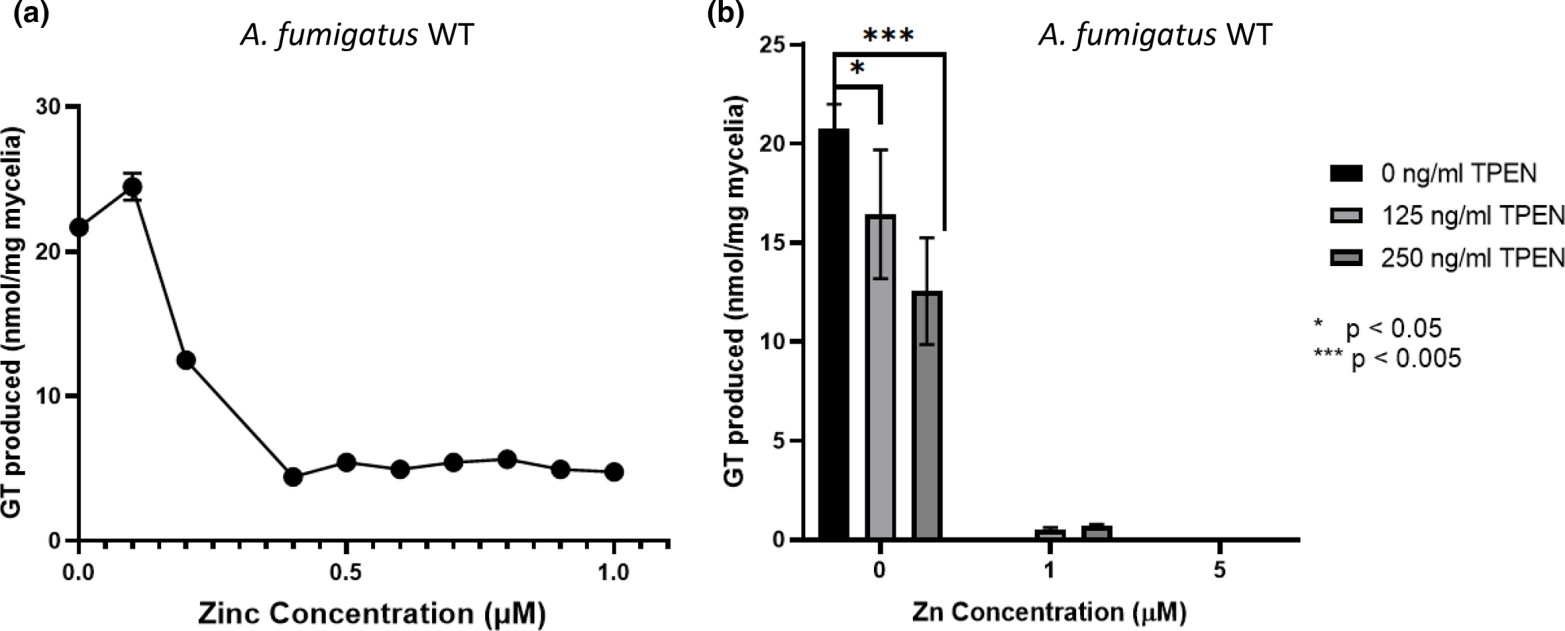
Extreme dependence of GT biosynthesis on low-level (nmol) Zn availability. RP-HPLC detection of GT produced by *A. fumigatus* wild-type. (**a**) Titration curve of GT production with increasing Zn between 0 and 1 µM. *n*=2. (**b**) Production of GT with varying TPEN and Zn concentrations in growth media. *n*=3.

To further investigate the relationship between increasing media zinc concentration and the decrease in GT production by *A. fumigatus* wild-type, a titration between 0 and 1 µM zinc in liquid culture was conducted. Compared to addition of 0 µM zinc, an initial increase in GT production by mycelia (nmol mg^−1^ mycelia) was seen in the presence of 0.1 µM zinc, although it dropped with further zinc addition (Fig. 3a). The amount of GT produced per mg mycelia reached steady-state at 0.5 µM zinc and remained stable as the zinc concentration was increased (Fig. 3a). These observations suggest that extreme zinc limitation (addition of 0 µM zinc) is sub-optimal for GT production, as it is for overall fungal fitness. To test the effect of added zinc-chelators on biosynthesis of GT, *A. fumigatus* wild-type was grown in the presence of TPEN (125 or 250 ng ml^−1^) with or without zinc. In zinc-depleted media, addition of TPEN significantly reduced the production of GT by 21 % at 125 ng ml^−1^ (*P=*0.04) and 40 % at 250 ng ml^−1^ (*P<*0.001). In the presence of 1 µM zinc, which is not permissive for endogenous GT production, both 125 and 250 ng ml^−1^ TPEN triggered very low levels of GT biosynthesis (0.5 and 0.7 nmol mg^−1^ mycelia, respectively) (Fig. 3b). This shows that zinc restriction by chelators in replete conditions stimulates GT production, while extreme zinc limitation (addition of 0 µM zinc) decreases the production of GT. These results also support the hypothesis that chelators may simulate a starved phenotype, upregulating processes involved in metal ion acquisition and uptake.

### TAFC production by *A. fumigatus* wild-type and Δ*gtmA* under low-iron conditions in response to increasing media zinc

**Fig. 4.**
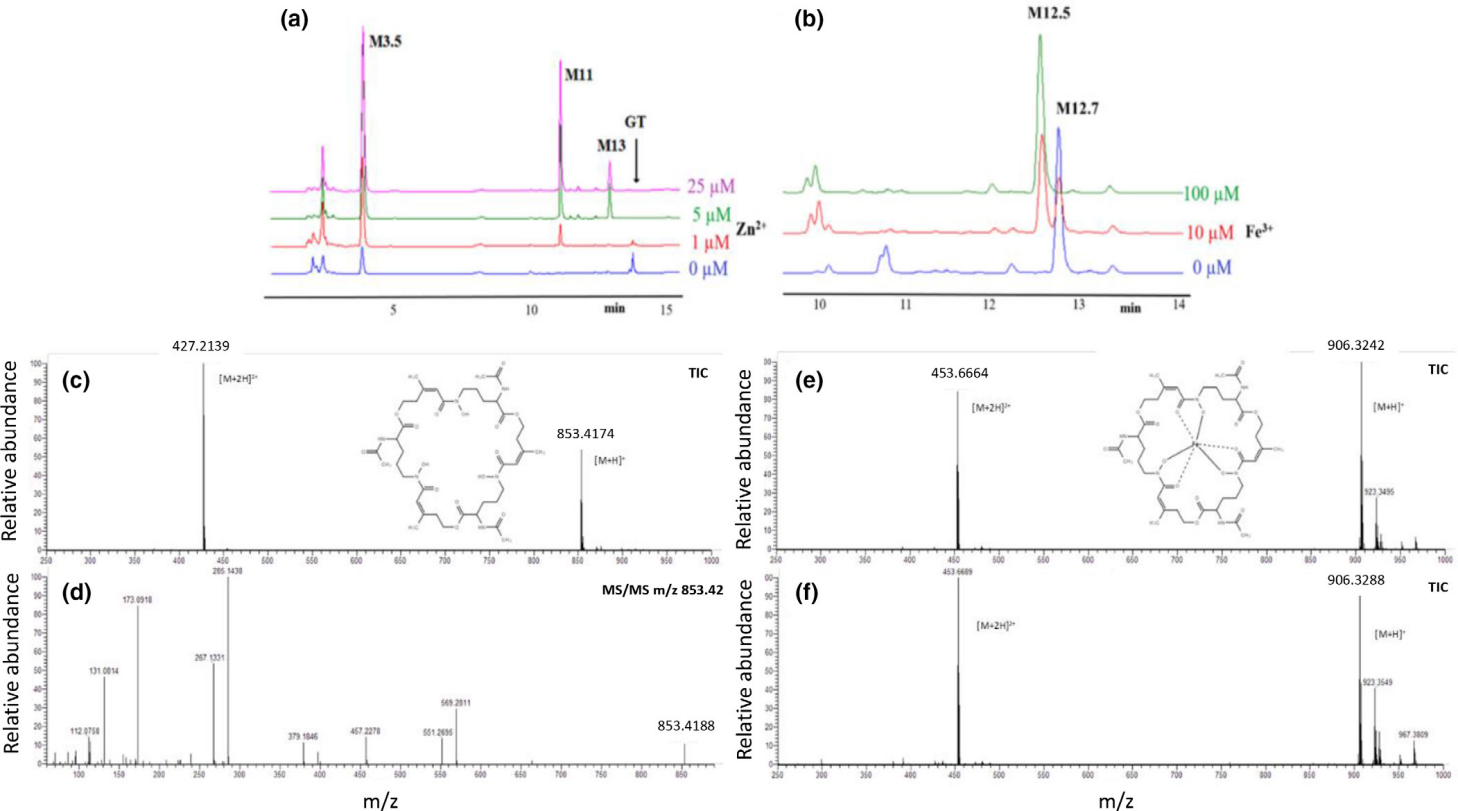
Triacetylfusarinine C (TAFC) secretion by *A. fumigatus* at high zinc concentrations and LC-MS identification. RP-HPLC profile of metabolites detected at 254 nm. (a) Wild-type profile of secreted metabolites from SDN low-iron culture supernatant. (b) Retention time shift of M12.7 (unferrated TAFC) to M12.5 (ferrated TAFC) with the addition of iron to collected supernatant. Identification of M13 in *A. fumigatus* zinc cultures as TAFC by LC-MS. (c) Mass spectra in positive ion mode of RP-HPLC fractionated peak M12.7 from wild-type zinc cultures [total ion chromatograph (TIC) and (d) tandem mass spectrum (MS/MS) of *m/z* 853.42 ion]. Unferrated TAFC equates to *m/z* 853.42 (structure inlay). (e) Comparison of ionization chromatographs of standard Fe [TAFC], *m/z*=906.32 (structure inlay) and (f) ferrated M12.5.

RP-HPLC analysis of *A. fumigatus* supernatant from increasing zinc concentration cultures showed a range of unidentified metabolites with retention times of 3.5 (M3.5), 11 (M11) and 12.7 min (termed M13) that increase with zinc addition (Fig. 4a). Wild-type cultures did not produce detectable amounts of M11.0 in the absence of zinc, while there was a low amount present in Δ*gtmA* cultures (Fig. S4b). Retention times of unidentified peaks were not influenced by reduction and alkylation. Post-secretion addition of FeSO_4_ (10 and 100 µM) shifted the retention time of peak M13 (precisely labelled as M12.7 in Fig. 4b) to 12.5 min and was detectable at 440 nm. Unexpectedly, M13 was identified as unferrated triacetylfusarinine C (TAFC), an iron-scavenging siderophore produced by *A. fumigatus* under iron-limiting conditions and identified by LC-MS [27] (Fig. 4c–f). TAFC was not produced by *A. fumigatus* wild-type at less than 5 µM zinc and production continued up to 25 µM zinc. Conversely, low levels of unferrated TAFC were detectable at 1 µM zinc for *A. fumigatus* Δ*gtmA*, with a significant increase (*P*=0.0063) in the amount produced at 5 µM zinc (Fig. S4a). These differences in metabolite profiles between the wild-type and Δ*gtmA* in the presence of varying zinc levels suggest that there is a differential response in metal ion sensing or regulation of metal-dependent processes, and that deletion of GtmA may influence more than just GT biosynthesis.

### Quantitative proteomics reveals new insight into zinc-responsive proteome of *A. fumigatus* wild-type and Δ*gtmA*

LFQ proteomics was used to identify changes to the *A. fumigatus* wild-type and Δ*gtmA* proteomes grown in zinc-depleted and zinc-replete (5 and 25 µM) conditions. Zinc availability resulted in significantly altered abundance (*P*<0.05) of 210 proteins from *A. fumigatus* wild-type (21.5 % of total proteins), compared to zinc-depleted growth conditions. Of these proteins, 38 % were decreased in abundance with zinc addition, 40 % were increased, 8 % were uniquely detectable in the absence of zinc, and the remaining 15 % were only detectable in the presence of zinc (Tables 1 and 2, S1 and S2). In contrast, the abundance of 454 proteins was altered in *A. fumigatus* Δ*gtmA* (25.1 % of total proteins) whereby zinc addition resulted in either reduced (35 %) or increased (26 %) abundance of proteins which underwent significant alteration (*P*<0.05). Moreover, 14 % of proteins were uniquely found in zinc-depleted conditions, while 25 % were only detectable under zinc-replete conditions (Tables 3 and 4, S3 and S4).

**Table 1.** Proteins with increased abundance (or unique) in *A. fumigatus* wild-type strains grown in Czapek-Dox media with zinc compared to without zinc Data are sorted by fold change, in descending order.

Protein name	Log_2_(fold increase)	*P*-value	Peptides	Sequence (%)	Protein ID
Aflatoxin B1-aldehyde reductase GliO-like, putative	Unique	n/a	11	49.1	AFUA_1G13370
Methyltransferase, putative	Unique	n/a	8	29	AFUA_2G14390
Isocyanide synthase **XanB**	Unique	n/a	76	72.9	AFUA_5G02660
Short-chain dehydrogenase/reductase family oxidoreductase, putative	Unique	n/a	16	46	AFUA_5G09290
*O*-Methyltransferase **GliM**	Unique	n/a	28	76.1	AFUA_6G09680
Oxidoreductase, 2OG-Fe(II) oxygenase family, putative	Unique	n/a	6	35.3	AFUA_8G00110
l-PSP endoribonuclease family protein, putative	4.62858	0.009149	11	89.3	AFUA_2G17470
*S*-(Hydroxymethyl)glutathione dehydrogenase	4.47342	0.008085	28	60.2	AFUA_2G01040
Hsp70 chaperone BiP/Kar2, putative	4.36206	0.001921	47	47.9	AFUA_2G04620
Cysteine-rich secreted protein	3.9371	0.001461	33	79.9	AFUA_7G01060
Uncharacterized; upregulated in conidia exposed to neutrophils	3.80331	0.001461	8	52.1	AFUA_1G09030
Glutathione peroxidase	3.72976	0.021992	23	46.4	AFUA_3G12270
Aromatic aminotransferase Aro8, putative	3.72432	0.004333	49	87.6	AFUA_2G13630
l-PSP endoribonuclease family protein (Hmf1), putative	3.5685	0.001461	11	60.1	AFUA_7G02340
12-Alpha,13-alpha-dihydroxyfumitremorgin C prenyltransferase **FtmH**	2.54403	0.007585	37	74.9	AFUA_8G00250
Isonitrile hydratase-like protein **XanA**	2.53522	0.010337	11	47.5	AFUA_5G02670
Iron/copper chaperone Atx1, putative	2.47668	0.009149	10	98.7	AFUA_1G08880
Gliotoxin *bis*-thiomethyltransferase **GtmA**	2.2811	0.027096	14	45.6	AFUA_2G11120
Histidine biosynthesis trifunctional protein	2.13292	0.021492	65	81.7	AFUA_1G14570
Verruculogen synthase **FtmF**	1.96603	0.009149	38	77.3	AFUA_8G00230
Cobalamin-independent methionine synthase **MetH/D**	1.21343	0.009149	83	83.7	AFUA_4G07360

**Table 2.** Proteins with decreased abundance in *A. fumigatus* wild-type strains grown in Czapek-Dox media with zinc compared to without zinc Data are sorted by fold change, in descending order.

Protein name	Log_2_ (fold decrease)	*P*-value	Peptides	Sequence (%)	Protein ID
Putative tyrosine decarboxylase, transcript induced by voriconazole	Unique	n/a	12	35	AFUA_2G04980
Alpha-mannosidase	Unique	n/a	53	45.9	AFUA_3G08200
l-Amino acid oxidase **LaoA**	Unique	n/a	22	40.5	AFUA_7G06810
Fatty-acyl coenzyme A oxidase (Pox1), putative	−6.75124	0.001980	30	51	AFUA_7G06090
Major allergen **Aspf2**	−5.16915	0.000768	24	58.3	AFUA_4G09580
Oxidoreductase, zinc-binding dehydrogenase family, putative	−4.13334	0.000982	22	78.8	AFUA_1G15610
NADH:flavin oxidoreductase/NADH oxidase family protein	−4.10428	0.005119	17	56	AFUA_2G04060
Proteasome endopeptidase complex	−3.6007	0.001980	24	91.3	AFUA_3G11300
Gliotoxin oxidoreductase **GliT**	−3.46731	0.005720	38	94.9	AFUA_6G09740
12-Oxophytodienoate reductase, putative	−3.39024	0.003051	14	46.7	AFUA_5G14330
Thioredoxin reductase	−3.27287	0.001762	21	74	AFUA_4G12990
Glutaminase, putative	−3.06718	0.000768	18	28.9	AFUA_3G10910
NADH-dependent flavin oxidoreductase, putative	−2.72911	0.001482	21	53.8	AFUA_7G06420
Catalase B **CatB/1**	−2.66263	0.003534	98	76.8	AFUA_3G02270
Oxidoreductase, short-chain dehydrogenase/reductase family	−2.40285	0.003956	5	34.3	AFUA_4G03680
Homocysteine synthase **CysD**	−1.97439	0.001482	22	56.2	AFUA_5G04250

**Table 3.** Proteins with increased abundance (or unique) in *A. fumigatus ΔgtmA* strains grown in Czapek-Dox media with zinc compared to without zinc Data are sorted by fold change, in descending order.

Protein name	Log_2_ (fold increase)	*P*-value	Peptides	Sequence (%)	Protein ID
Oxidoreductase, short-chain dehydrogenase/reductase family	Unique	n/a	7	34.3	AFUA_4G03680
Allergen **Aspf7**	Unique	n/a	3	15.6	AFUA_4G06670
Zinc metallopeptidase, putative	Unique	n/a	7	26.6	AFUA_6G09600
l-Amino acid oxidase **LaoA**	Unique	n/a	22	40.5	AFUA_7G06810
Pyruvate carboxylase	3.55745	0.006854	73	69.7	AFUA_4G07710
Glycerol kinase, putative	3.49014	0.001297	26	50.9	AFUA_6G08470
Probable beta-glucosidase A **BglA**	3.21015	0.000732	29	33.5	AFUA_1G05770
Major allergen **Aspf2**	3.20795	0.006288	24	58.3	AFUA_4G09580
Peptidyl-arginine deiminase domain protein	3.18142	0.006223	36	79.4	AFUA_8G06520
6-Hydroxytryprostatin B *O*-methyltransferase **FtmD**	2.99338	0.001055	55	87.6	AFUA_8G00200
Catalase B **CatB/1**	2.43172	0.002146	98	76.8	AFUA_3G02270
Superoxide dismutase [Mn], mitochondrial (allergen Aspf6) **SodC**	2.42899	0.037797	31	97.1	AFUA_1G14550
Histidine biosynthesis trifunctional protein	2.2504	0.001919	65	81.7	AFUA_1G14570
Iron/copper chaperone **Atx1**, putative	2.08295	0.043061	10	98.7	AFUA_1G08880
Gliotoxin oxidoreductase **GliT**	1.61891	0.002884	38	94.9	AFUA_6G09740
12-Alpha,13-alpha-dihydroxyfumitremorgin C prenyltransferase **FtmH** [48]	1.4838	0.015104	37	74.9	AFUA_8G00250
Catalase A **CatA**	1.33686	0.012140	72	86.1	AFUA_6G03890
Oxidoreductase, zinc-binding dehydrogenase family, putative	1.12874	0.040982	22	78.8	AFUA_1G15610
Tryprostatin B synthase **FtmPT2**	1.0477	0.015104	37	74.9	AFUA_8G00250

**Table 4.** Proteins with decreased abundance in *A. fumigatus ΔgtmA* strains grown in Czapek-Dox media with zinc compared to without zinc Data are sorted by fold change, in descending order.

Protein name	Log_2_ (fold decrease)	*P*-value	Peptides	Sequence (%)	Protein ID
Zn-dependent hydrolases of the beta-lactamase fold, putative	Unique	n/a	7	42.3	AFUA_1G01460
Aromatic hydroxylase **FmpF**	Unique	n/a	25	53	AFUA_6G03490
Multifunctional cytochrome P450 monooxygenase **FmaG**	Unique	n/a	6	20	AFUA_8G00510
Alpha/beta hydrolase **PsoB**	Unique	n/a	12	28.3	AFUA_8G00530
Amino acid oxidase **FmpA**	−7.85265	0.000558	43	85.5	AFUA_6G03440
Fumipyrrole biosynthesis protein C **FmpC**	−6.3108	0.006005	17	33.3	AFUA_6G03460
PKS-NRPS hybrid synthetase **PsoA** [49]	−5.52392	0.000525	4	42.8	AFUA_8G00540
Isocyanide synthase **XanB**	−4.10497	0.031579	76	72.9	AFUA_5G02660
*O*-Methyltransferase **GliM**	−3.59461	0.010986	28	76.1	AFUA_6G09680
Extracellular metalloproteinase **Mep** (allergen Aspf5)	−3.32339	0.009946	16	38.5	AFUA_8G07080
Toxin biosynthesis protein **GliH**, putative	−3.0969	0.000581	20	82.1	AFUA_6G09745
Allergen **Aspf1**	−2.89312	0.000665	10	67	AFUA_5G02330
Cytochrome P450 monooxygenase **GliF**	−2.76922	0.006688	19	32.7	AFUA_6G09730
Isonitrile hydratase-like protein **XanA**	−2.41018	0.017907	11	47.5	AFUA_5G02670
Polyketide transferase af380 **FmaC**	−2.39468	0.007973	24	93.2	AFUA_8G00380
*N*-Methyltransferase **GliN**	−2.30887	0.008274	36	99.6	AFUA_6G09720
Aflatoxin B1-aldehyde reductase GliO-like, putative	−2.15074	0.007603	11	49.1	AFUA_1G13370
Dioxygenase af480 **FmaF**	−1.97978	0.001868	7	30.1	AFUA_8G00480
Homocysteine synthase **CysD**	−1.86178	0.000906	22	56.2	AFUA_5G04250
Dual-functional monooxygenase/ methyltransferase **PsoF**	−1.65219	0.005662	31	43.9	AFUA_8G00440
Methyltransferase **PsoC**	−1.33021	0.002108	42	81.5	AFUA_8G00550
Glutathione *S*-transferase **PsoE**	−1.16869	0.020172	15	51.1	AFUA_8G00580

### Altered zinc-responsiveness in *A. fumigatus* Δ*gtmA*

From a consistency and comparability perspective, several observed proteomic responses of *A. fumigatus* wild-type agree with previously reported genomic studies in response to zinc [8, 28, 29]. Vicentefranqueira *et al.* [8] revealed that genes encoding Aspf2, CatB/Cat1, LaoA/SarA and GliT were induced directly by ZafA and are therefore expressed under zinc-limiting conditions. Correspondingly, our results show that Aspf2, CatB/Cat1, LaoA/SarA and GliT abundances are attenuated by zinc addition. Reduced abundance of the GT self-protection enzyme GliT following addition of zinc to cultures (Table 2) also accords with previously observed transcriptomic experimental data. A decrease in GliT abundance is also supported by RP-HPLC data where levels of GT are decreased at higher concentrations of zinc in growth media. However, the deletion strain *A. fumigatus* Δ*gtmA* exhibited a different response to increasing zinc levels. Aspf2, CatB/Cat1, LaoA/SarA and GliT abundance were all significantly increased in zinc-replete compared to zinc-depleted conditions. The pattern of GliT abundance is probably due to the deletion of GtmA and the subsequent loss of the GT downregulatory mechanism of *A. fumigatus* and is matched by RP-HPLC observations. Vicentefranqueira *et al.* [8] showed that *sodC* expression is directly regulated by ZafA and is induced by the transcription factor under zinc-limiting conditions. We observed the opposite trend in *A. fumigatus* Δ*gtmA* where SodC abundance was increased with the addition of zinc (Table 3). The observed changes in abundance in the Δ*gtmA* mutant suggest that there may be altered zinc-sensing in this mutant.

**Fig. 5.**
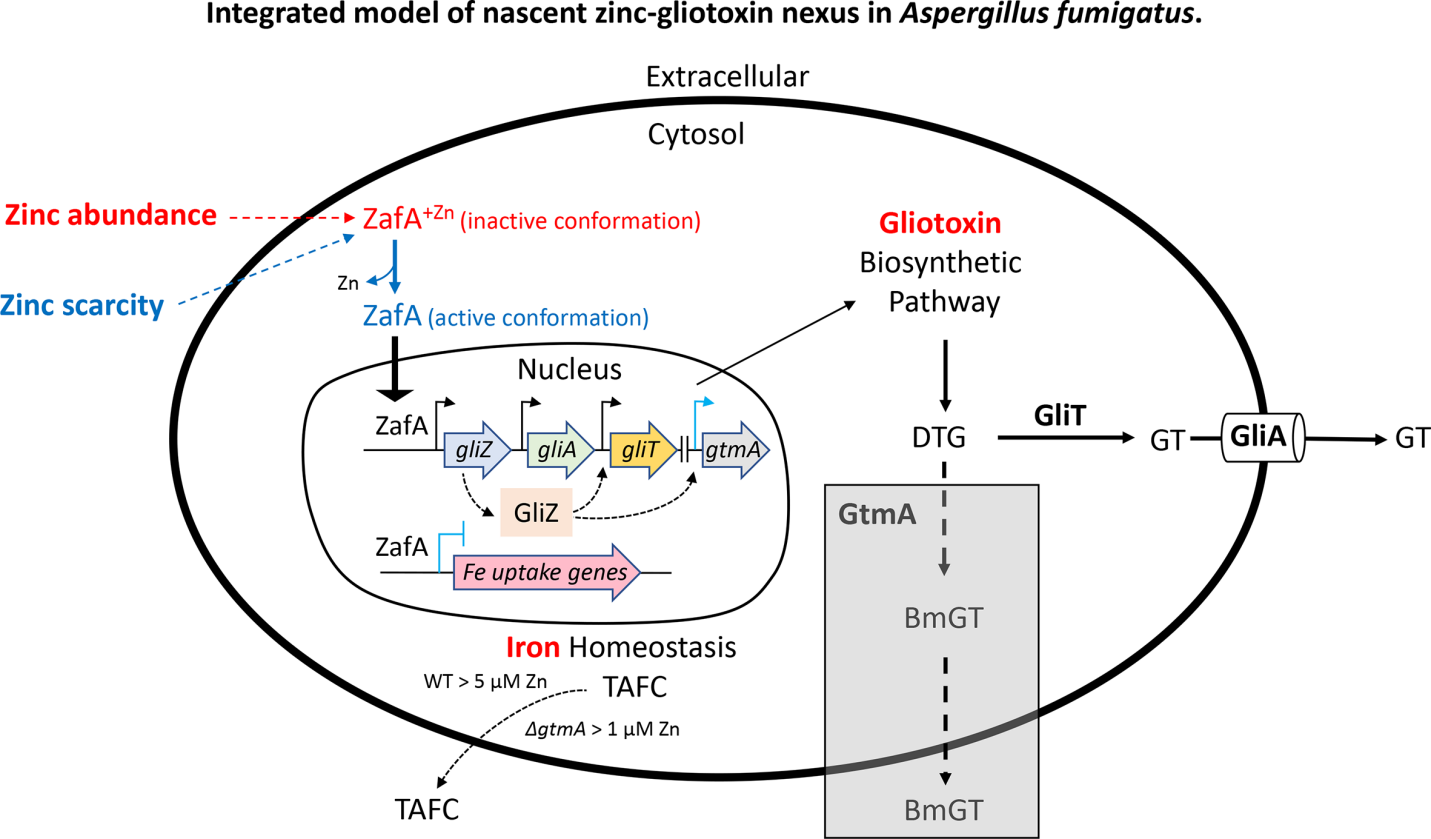
Integrated model of nascent zinc–gliotoxin nexus in *A. fumigatus*. Zinc absence or restriction promotes ZafA to enter the nucleus, and functions with GliZ to drive *gli* biosynthetic gene cluster expression and gliotoxin biosynthesis/secretion. Shaded region: GtmA normally dissipates GT production by alternative BmGT secretion [50]. However, in the presence of zinc and in the absence of GtmA, GT biosynthesis cannot be attenuated, which results in elevated GliT, continued GT secretion and premature activation of siderophore secretion to facilitate Fe^3+^ uptake to maintain intracellular Zn:Fe homeostasis [38].

Proteomic analysis also showed a change in the number of proteins with oxidoreductase activity in *A. fumigatus* Δ*gtmA* compared to the wild-type. In the wild-type, 26 proteins were significantly altered in abundance with zinc addition, while the abundance of 48 proteins was changed significantly in the mutant. Zinc addition decreased the abundance of 17 wild-type proteins and increased the abundance of nine. In *A. fumigatus* Δ*gtmA,* 26 proteins were decreased in abundance with zinc addition while 22 were increased in abundance. Several of the proteins with altered abundance with zinc addition have inverse responses in the wild-type and mutant. Two proteins which were seen to be increased in abundance in the wild-type were decreased in the GtmA-deficient mutant, while six proteins found to be decreased in the wild-type with zinc addition were correspondingly increased in abundance with the addition of zinc to *A. fumigatus* Δ*gtmA*, including CatB/1 and GliT (Table 5). The deletion of *gtmA* from *A. fumigatus* results in a shift towards GliT oxidation of intracellular DTG for subsequent GliA-mediated efflux from mycelia (Fig. 5). An increase in GT redox cycling alongside altered zinc-sensing contribute to the differential proteomic response of *A. fumigatus* Δ*gtmA* to zinc availability in growth media.

**Table 5.** Log_2_-fold change of proteins differentially altered in abundance with zinc addition in the *A. fumigatus* wild-type and Δ*gtmA* datasets

Protein ID	Log_2_ fold change	
	Wild-type	*ΔgtmA*
**Increased in wild-type, decreased in mutant**		
Aflatoxin B1-aldehyde reductase GliO-like	Unique to Zn presence	−2.151
Amine oxidase	1.223	−2.656
**Decreased in wild-type, increased in mutant**		
l-Amino acid oxidase **LaoA**	Unique to Zn absence	Unique to Zn presence
Oxidoreductase, zinc-binding dehydrogenase family	−4.133	1.129
Gliotoxin oxidoreductase **GliT**	−3.467	1.619
Catalase B **CatB/1**	−2.663	2.432
Oxidoreductase, short-chain dehydrogenase/reductase family	−2.403	Unique to Zn Presence
2-Nitropropane dioxygenase family oxidoreductase	−2.146	1.018

Secondary metabolism also appears to be altered in *A. fumigatus* Δ*gtmA* compared to the wild-type in response to changing zinc availability. Two proteins from the *xan* biosynthetic gene cluster (BGC), XanA and XanB, were found to be increased in abundance with zinc and unique to zinc-replete conditions respectively in *A. fumigatus* wild-type cultures (Table 1). However, both proteins were subsequently found to be decreased in abundance in response to zinc addition in the *gtmA*-deletion mutant (Table 4). The *xan* BGC is involved in the production of the isocyanide xanthocillin and has been shown to be copper-responsive [30]. Altered zinc sensing capabilities of *A. fumigatus* Δ*gtmA* and corresponding changes in zinc homeostasis and regulation may influence the regulation and response to other transition metals, such as copper and iron. Several other BGCs were influenced by the addition of zinc to culture media. Proteins involved in the biosynthesis of fumitremorgin-type indole alkaloids were increased in abundance with the addition of zinc in both strains. Meanwhile multiple proteins from the fumipyrrole BGC (three) and intertwined fumagillin/pseurotin supercluster (eight) were found to be decreased in abundance or unique to zinc-limiting conditions for *A. fumigatus* Δ*gtmA*. The differential response of the wild-type and GtmA-deficient proteome to changing zinc concentrations in media, alongside changes in the secreted metabolome, not only shows that zinc availability plays a role in the production of different proteins from multiple pathways and processes, but also suggests that the inability to regulate GT biosynthesis via BmGT formation has wider reaching effects on *A. fumigatus*. Finally, increased abundance of an iron/copper chaperone Atx1 was detected in *A. fumigatus* Δ*gtmA* lysates upon zinc supplementation of CD media (Table 3).

## Discussion

Here we provide evidence that zinc availability and GT biosynthesis in *A. fumigatus* are intrinsically linked. We demonstrate that the growth-inhibitory effects of exogenous GT are relieved by the presence of zinc in growth media, and that higher concentrations of GT and TPEN are required to produce *A. fumigatus* growth inhibition on nutrient-rich media. We also reveal that the production of GT by *A. fumigatus* is negatively influenced by the presence of zinc, and that zinc differentially alters the secreted metabolome of *A. fumigatus* wild-type and Δ*gtmA*, respectively, the latter deficient in BmGT biosynthesis. Furthermore, LFQ proteomics reveals differences in the proteomic response of these strains to the presence or absence of zinc. Overall, our observations suggest that the interactions between GT and zinc in *A. fumigatus* play an important role in cellular function, and the loss of BmGT formation may have further impact beyond regulation of GT biosynthesis, possibly mediated by zinc sensing (Fig. 5).

Solid and liquid culture growth assays show the hypersensitivity of *A. fumigatus* Δ*gliT*Δ*gtmA* to exogenous GT and the restoration of growth by zinc addition to media. It was previously shown that *A. fumigatus* Δ*gliT*Δ*gtmA* displayed a greater susceptibility to exogenous GT-mediated growth inhibition than either *A. fumigatus* Δ*gliT* or Δ*gtmA* alone [24]. Moreover, Saleh *et al.* [3] revealed that zinc addition alongside exogenous GT restored growth of the double mutant. That work was conducted on CD agar, which is a zinc-limiting but not completely Zn-deficient medium, which is permissive for endogenous GT biosynthesis. We observed the same trend of growth inhibition of *A. fumigatus* Δ*gliT*Δ*gtmA* by GT, and subsequent zinc relief, but this time under controlled zinc conditions. Thus, the proposed model for observed growth inhibition is zinc-chelation and inactivation of zinc-dependent enzymes by intracellular DTG. Exogenous GT stimulates further biosynthesis by inducing *gli* cluster activation [31] and the absence of both the self-protection oxidoreductase GliT and negative regulator GtmA leads to accumulation of DTG in *A. fumigatus* Δ*gliT*Δ*gtmA*.

Interestingly, higher concentrations of exogenous GT are required to cause the same growth inhibition on SAB as on SDNE, which is explained by higher zinc levels in the former growth medium [32, 33]. TPEN also inhibits the growth of both *A. fumigatus* wild-type and Δ*gliT*Δ*gtmA* on SDNE and SAB medium, with the double mutant revealing a higher sensitivity to TPEN presence. One possible reason for this is co-inhibition of growth by TPEN and endogenous DTG produced in response to zinc-starvation by TPEN. It was also noted that higher concentrations of TPEN were required to cause growth inhibition on SAB compared to SDNE due to higher zinc in the medium. TPEN has previously shown inhibitory effects against *Candida albicans* and *A. fumigatus*, and the presence of zinc blocks this inhibitory activity against both fungi [34, 35]. Laskaris *et al.* [34] showed that TPEN also augments the activity of antifungal caspofungin against *A. fumigatus* and increases the survival of infected mice. Further work is required to investigate the combinatorial impact of TPEN and GT against *A. fumigatus* and the influence of zinc on these growth conditions.

Saleh *et al.* [3] and Vicentefranqueira *et al.* [8] first noted inhibition of GT biosynthesis by zinc and the influence of ZafA on *gli* BGC expression, respectively, with similar observations by Seo *et al.* [36]. In the present work, production of GT by *A. fumigatus* was inhibited by the presence of nanomolar zinc, whereby *A. fumigatus* wild-type produces 21 nmol GT per mg of mycelia in zinc-free media. The addition of low concentrations of zinc (>0.4 µM) to growth media reduces GT production, showing that an effective absence (0–4 µM) of zinc is required for biosynthesis. This observation is in accordance with transcriptomic data where *gli* cluster genes were found to be under the control of ZafA, the transcription factor responsible for regulation of zinc homeostasis [8]. Expression levels of *gliZ* (the zinc finger transcription factor which regulates *gli* expression [37]), *gliT*, *gliA* and *gtmA* were all strongly induced under zinc-limiting conditions compared to 100 µM zinc. Although not formally shown, all previous experimental evidence strongly suggests that, in zinc-replete media, the putative zinc binding domains located in the N-terminal region of the ZafA transcription factor became saturated with zinc ions such that ZafA would adopt a regulatory inactive conformation, unable to bind DNA, that would be also unable to enter the nucleus. In contrast, under zinc-limiting conditions ZafA is able to enter the nucleus and bind to DNA to regulate gene expression [38] (Fig. 5). On the other hand, given that ZafA is a major inducer of *gliZ* expression under zinc-limiting conditions [8], ZafA inactivation in the presence of zinc would result in a strong reduction of the *gliZ* expression level and, hence, in GT biosynthesis [36]. The influence of zinc on GT biosynthesis suggests that under zinc-limiting conditions *A. fumigatus* produces GT to assist growth and proliferation, or possibly to acquire zinc to overcome zinc-limited growth. The connection between concurrent zinc inhibition of GT production and exogenous GT upregulating *de novo* biosynthesis remains an open question [4]. The switch from GT biosynthesis to BmGT biosynthesis with low micromolar zinc availability further strengthens the connection between zinc and GT. It also highlights a role for GtmA in this response. Interestingly, Danchin [39] has speculated on a global metabolic integration role for zinc.

The extracellular metabolite profile of *A. fumigatus* Δ*gtmA* in response to zinc is different from that of the wild-type. Compared to the wild-type strain, *A. fumigatus* Δ*gtmA* produced less GT under zinc-limited conditions, which was unexpected, as loss of GtmA-catalysed BmGT formation should augment the GT-positive feedback system [23]. However, the continued production of GT with increasing zinc availability in this mutant links zinc and GtmA-mediated negative regulation of GT biosynthesis. Conceivably, the inability to switch off GT biosynthesis impacts zinc-sensing or zinc-regulation in *A. fumigatus*. Previously, Doyle *et al.* [22] showed that dysregulated GT biosynthesis via deletion of *gtmA* influences secondary metabolite production in long-term cultures. In particular, pseurotin A, fumagillin and fumitremorgin biosynthesis was affected. These previous experiments were conducted in CD media without the context of a zinc influence on GT cluster activation and production. The observation of TAFC production by *A. fumigatus* wild-type and Δ*gtmA* strains in zinc cultures was unexpected. The addition of 1 µM zinc to media activated TAFC production in *A. fumigatus* Δ*gtmA* but not in the wild-type. At 5 µM and higher, TAFC was produced in both strains. This observation supports the hypothesis that loss of GT-negative regulation impacts the zinc response of *A. fumigatus* in culture. The relationship between iron and zinc homeostasis in *A. fumigatus* has been previously reported. Vicentefranqueira *et al.* [38] showed that the ratio of iron to zinc in culture media influences the regulation of ion homeostasis, and also determines fungal growth capacity, which is also supported by increased abundance of a putative iron/copper transporter Atx1 in our proteomic work. Yasmin *et al.* [40] revealed that iron starvation increases zinc toxicity and alters the regulation of zinc uptake.

The zinc-responsive proteome of *A. fumigatus* may provide further insight into pathways or mechanisms required for fungal survival during host infection. An altered abundance of specific proteins, with increasing zinc concentrations, suggests that they may play a role during zinc limitation. Aspf2 is a major allergen associated with *A. fumigatus* under direct regulation of ZafA and is required for growth under extreme zinc-limited conditions, in particular alkaline conditions [28]. It has been shown to bind IgE in patients with allergic bronchopulmonary aspergillosis (ABPA), and complement system regulators Factor-H and Factor-H-like-protein (FHLP-1) in immunocompromised patients [41, 42]. The observed reduction of Aspf2 abundance with addition of zinc to *A. fumigatus* wild-type agrees with previous literature and confirms the importance of this protein during zinc starvation. Interestingly, the abundance of Aspf2 was significantly increased in *A. fumigatus* Δ*gliT*Δ*gtmA* compared to *A. fumigatus* Δ*gliT* in response to exogenous GT, implying greater zinc restriction [24]. An abundance of the homocysteine synthase CysD by the wild-type was reduced with the addition of 25 µM zinc. Of relevance, Sugui *et al.* [43] have shown that *cysD* expression was upregulated upon exposure to human neutrophils, implying zinc restriction. Neutrophils produce extracellular traps (NETs) and secrete high levels of the zinc- and manganese-binding protein calprotectin during infection. A lower abundance of GliT with addition of zinc supports the model that zinc availability abrogates GT production. GliT is required for the protection against intracellular DTG toxicity in *A. fumigatus*, particularly under zinc-limiting conditions. GT biosynthesis is controlled by GliZ [44], which itself is directly regulated by ZafA.

Elevated abundance of zinc-dependent methionine synthase MetH/D was found with addition of 25 µM zinc, and was also found to be induced by heat shock in *A. fumigatus* [45], is either produced at high zinc due to an activity requirement, or is repressed under zinc-starved conditions. Interestingly there are strong links between the methyl/methionine cycle and GT biosynthesis via the utilization of SAM [46], and the conditional essentiality of MetH has recently been demonstrated [47].

GtmA-deficient *A. fumigatus* has an altered zinc response to the wild-type. Differences were observed between the secreted metabolite profile of *A. fumigatus* wild-type and Δ*gtmA* grown in zinc-deplete and zinc-replete conditions, and changes in proteomic responses to zinc supports our hypothesis that loss of GtmA influences the zinc response and sensitivity. Proteins involved in the biosynthesis of several secondary metabolites are uniquely present in zinc-limiting conditions or have decreased abundance with the addition of zinc. Two secondary metabolites which are negatively influenced by zinc addition are pseurotin A and fumagillin. Five and four proteins involved in biosynthesis, respectively, are reduced in abundance in zinc-replete cultures, with one from each cluster is expressed only in the absence of zinc. Another secondary metabolite biosynthetic process which is altered with zinc is that encoding the isocyanide xanthocillin [30]. A decreased abundance of both XanA and XanB in zinc-replete mutant cultures suggests production of xanthocillin in the absence of zinc. However, the opposite is observed in the wild-type. Both proteins observed to be decreased in abundance in *A. fumigatus* Δ*gtmA* were reciprocally affected with zinc addition to the wild-type. This inverse response supports the altered zinc-response model of the BmGT-deficient mutant, *A. fumigatus* Δ*gtmA*.

The negative impact of zinc on GT biosynthesis has been observed at the proteomic level also, whereby increasing zinc in growth media decreased the abundance of four *gli* cluster proteins in the mutant, in accordance with RP-HPLC observations. However, in *A. fumigatus* Δ*gtmA*, the abundance of GliT increases in response to 5 µM zinc, the opposite to the wild-type response. The increased abundance of GliT may be a direct response to the absence of GtmA in an attempt by the fungus to prepare for GT oxidation/detoxification. Saleh *et al.* [3] revealed that zinc prevented the modification of DTG thiols by GtmA-mediated methylation or chemical alkylation with IAA. It is likely that zinc chelation also impairs the oxidation of DTG to GT by GliT, which may also account for increased abundance of GliT in zinc-replete mutant cultures. Unexpectedly, an abundance of the major allergen Aspf2 increased with zinc addition to *A. fumigatus* Δ*gtmA*, in contrast to the decrease observed in the wild-type. This was not the only ZafA-regulated protein found to be increased in abundance in zinc-replete cultures. Increased abundance of proteins involved in oxidation–reduction processes was also observed. Levels of both CatA, a fast catalase [15], and CatB/Cat1 were elevated in zinc-replete cultures. The change in CatB/Cat1 abundance is the opposite to that in the wild-type, where added zinc decreased protein abundance. Similarly, there was an increase in the level of the SodC superoxide dismutase and the l-amino acid oxidase LaoA/SarA in mutant zinc-replete cultures. Like *catB/cat1* and *aspf2*, expression of *sodC* and *laoA/sarA* was found to be directly regulated by ZafA in Vicentefranquiera *et al.* [8] and should be repressed by zinc. The increased abundance of these proteins in *A. fumigatus* Δ*gtmA* suggests a possible zinc-sensing deficiency. It also suggests that the presence of zinc augments mutant sensitivity to ROS stress or altered levels of ROS production compared to the wild-type. Interestingly, Owens *et al.* [21] found that GT alleviates H_2_O_2_-induced oxidative stress by acting as an antioxidant, so a lack of GT production in zinc-replete cultures may infer increased sensitivity to oxidative stress.

## Conclusions

Overall, our observations show that (i) intracellular zinc chelation by TPEN and GT impedes fungal growth, (ii) available zinc directly influences both GT and BmGT biosynthesis, and that of unrelated metabolites, and finally (iii) zinc-dependent differential proteomic responses are evident between *A. fumigatus* wild-type and Δ*gtmA*. This reveals an unforeseen relationship between metal ion homeostasis and BGC-encoded activity (Fig. 5). Given the importance of zinc acquisition for fungal virulence, our observations may lead to further revelation of microbial BGC functionality and involvement in overcoming nutritional immunity, in the struggle between host and pathogen to retain and exploit zinc.

## Supplementary Data

Supplementary material 1
