# Supplementary material for: At the metal–metabolite interface in Aspergillus fumigatus: towards untangling the intersecting roles of zinc and gliotoxin
Source: Microbiology (Reading). 2021 Nov 5;167(11):001106. doi: 10.1099/mic.0.001106 (PMC8743625; doi:10.1099/mic.0.001106)
Supplement: Supplementary material 1 [file mic-167-1106-s001.pdf]

At the metal-metabolite interface in *Aspergillus fumigatus*: towards untangling the intersecting roles of zinc and gliotoxin.

Aimee M. Traynor[1], Rebecca A. Owens[1], Claudia M. Coughlin[1], Maeve C. Holton[1], Gary W. Jones[2], José A. Calera[3,4] and Sean Doyle[1].

[1] Department of Biology, Maynooth University, Maynooth, Co. Kildare, Ireland.
[2] Centre for Biomedical Science Research, School of Clinical and Applied Sciences, Leeds Beckett University, Leeds, United Kingdom.
[3] Instituto de Biología Funcional y Genómica (IBFG-CSIC), Universidad de Salamanca, Salamanca, Spain.
[4] Departamento de Microbiología y Genética, Universidad de Salamanca, Salamanca, Spain.

* Corresponding author:

Professor Sean Doyle, Department of Biology, Maynooth University, Maynooth, Co. Kildare, Ireland.

Email: sean.doyle@mu.ie; Tel : +353-1-7083858

Keywords: BGC, nutritional immunity, gliotoxin, zinc, quantitative proteomics, fungal drug targets.

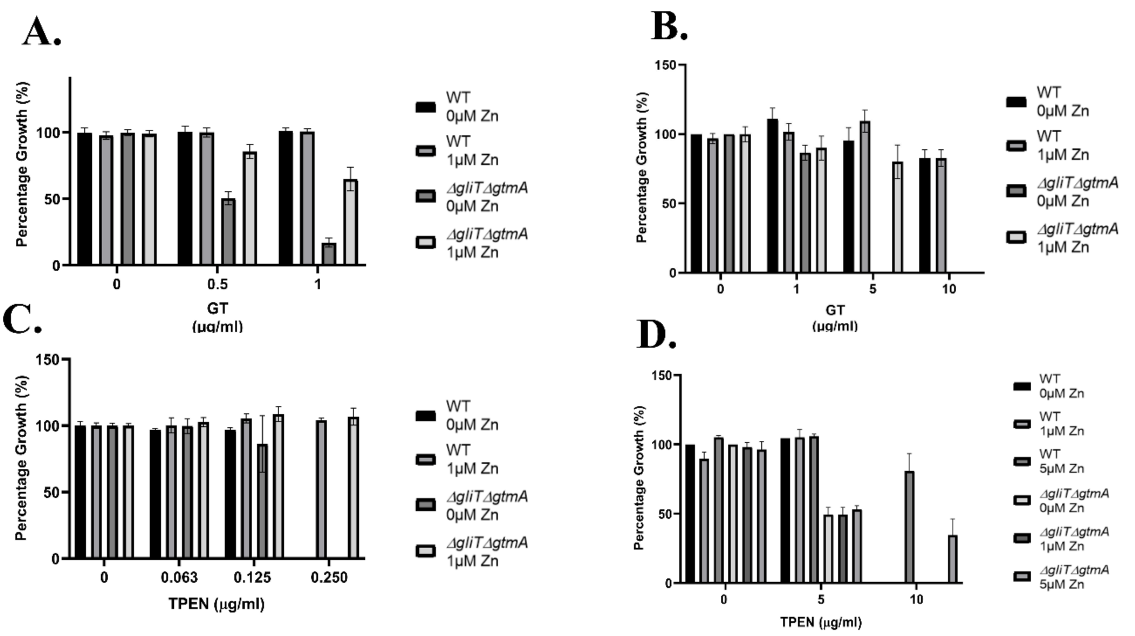

Supplementary Figure S1. Growth inhibition assays of *A. fumigatus* wild type and Δ*gliT*Δ*gtmA* on solid-state agar showing mutant hypersensitivity to GT and increased sensitivity to TPEN compared to wild-type. Absence of bars represents zero radial growth on solid-state agar. **A.** GT mediated growth inhibition against *A. fumigatus* on SDNE agar with or without added zinc. **B.** GT mediated growth inhibition against *A. fumigatus* on SAB agar with or without zinc addition. **C.** *A. fumigatus* growth inhibition on SDNE in the presence of TPEN with or without addition of zinc. **D.** Inhibition of *A. fumigatus* growth on SAB in the presence of TPEN with or without supplemented zinc. n = 3.

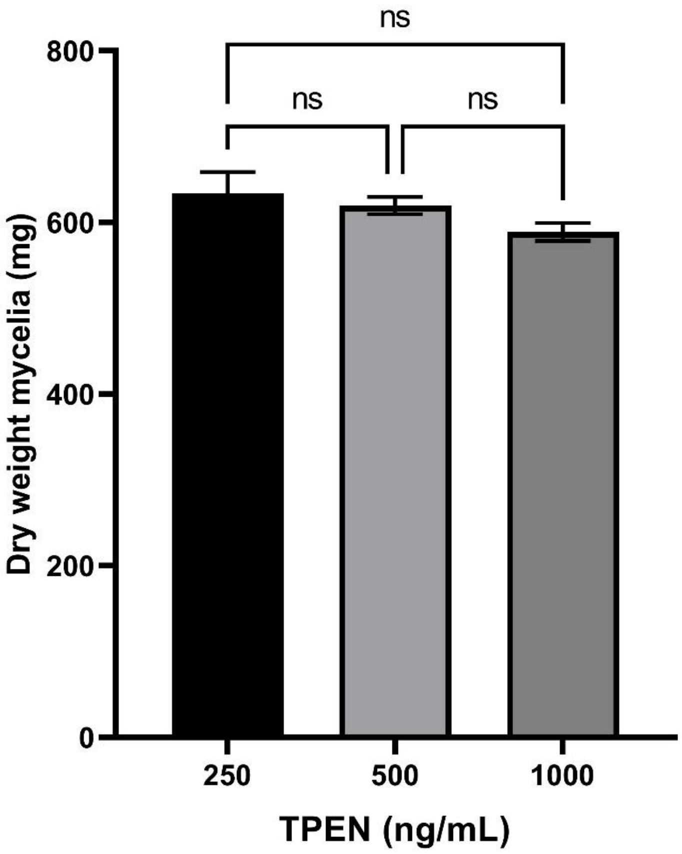

Supplementary Figure S2. Addition of higher concentrations of TPEN (500 ng/ml and 1000 ng/ml) to *A. fumigatus* Δ*gliT*Δ*gtmA* with 5 µM zinc does not further increase the mycelial mass. Observed decrease in growth is non-significant (ns). n = 3.

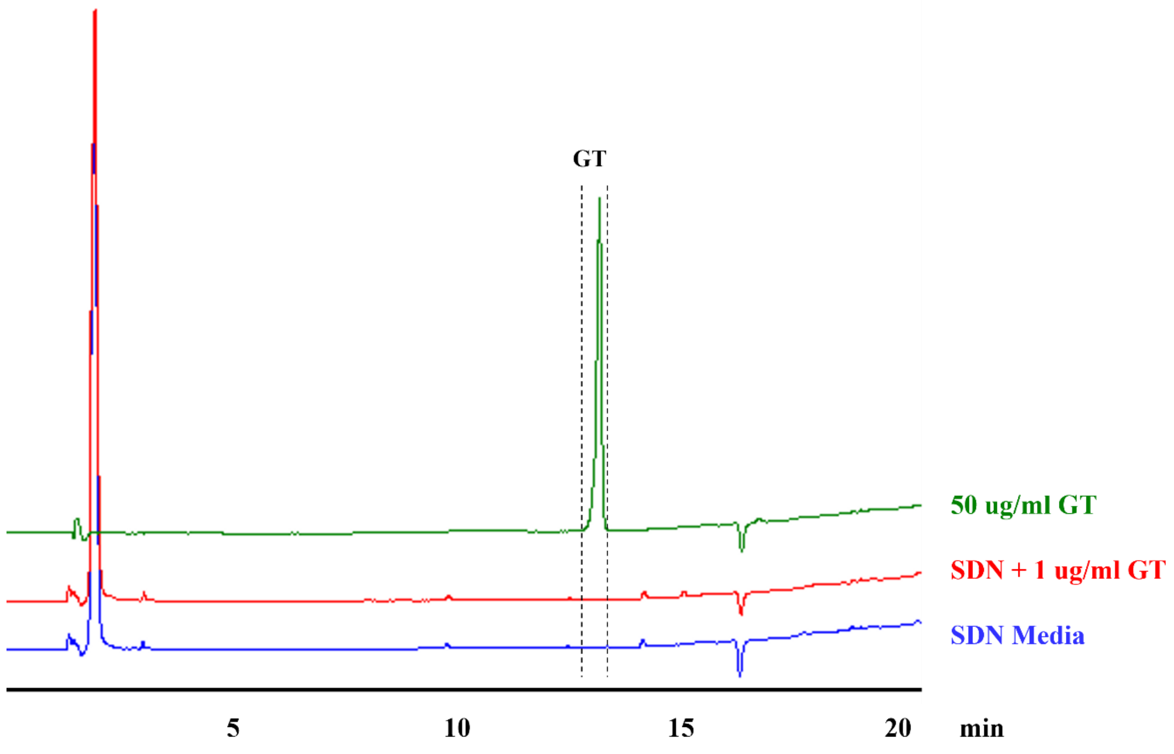

Supplementary Figure S3. RP-HPLC chromatograms comparing SDN media with no added gliotoxin (GT) in blue (bottom line), SDN media with 1 µg/ml exogenous GT in red (middle line), and a GT standard at a concentration of 50 µg/ml as a reference time in green (top line). The low concentration of exogenous GT (1 µg/ml) added to growth media is not detectable by RP-HPLC.

A.

B.

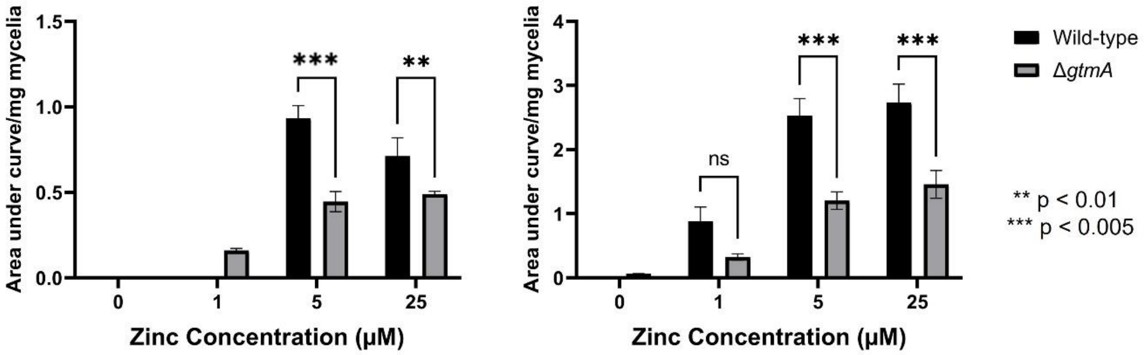

Supplementary Figure S4. *A. fumigatus* wild-type and Δ*gtmA* differentially produce **A.** TAFC and **B.** M11 in response to zinc addition to low iron SDN liquid medium. RP-HPLC measurements of area under the curve (mAU) per mg of mycelia. n = 3.

Supplementary Table S1: Proteins with increased abundance (or unique) in *A. fumigatus* wild-type strains grown in Czapek-Dox media with zinc compared to without zinc. Data sorted by fold change, in descending order.

Protein Description	Log2-fold Increase	P-value	No. of peptides	Sequence coverage (%)	Protein IDs AFUA_
ATP synthase delta chain, mitochondrial, putative	Unique to Zn Presence	n/a	7	55.2	1G03100
CBS domain protein	Unique to Zn Presence	n/a	15	42.9	1G06660
Protein phophatase 2C family protein	Unique to Zn Presence	n/a	7	16.6	1G06860
GPI anchored protein, putative	Unique to Zn Presence	n/a	6	23	1G10590
Thioesterase family protein	Unique to Zn Presence	n/a	3	14.5	1G10800
Aflatoxin B1-aldehyde reductase GliO-like, putative	Unique to Zn Presence	n/a	11	49.1	1G13370
tRNAHis guanylyltransferase Thg1, putative	Unique to Zn Presence	n/a	5	15.4	1G14630
Thiamine biosynthetic bifunctional enzyme, putative	Unique to Zn Presence	n/a	9	22.7	2G08970
NADH-ubiquinone dehydrogenase 24 kDa subunit, putative	Unique to Zn Presence	n/a	6	28	2G09130
Protein arginine methyltransferase NDUFAF7	Unique to Zn Presence	n/a	6	15.5	2G09740
Methyltransferase, putative	Unique to Zn Presence	n/a	8	29	2G14390
26S proteasome regulatory subunit S5A	Unique to Zn Presence	n/a	6	34.3	2G15070
Protein-L-isoaspartate O-methyltransferase	Unique to Zn Presence	n/a	8	64.2	3G05390
Acyl-CoA:6-aminopenicillanic-acid-acyltransferase, putative	Unique to Zn Presence	n/a	21	74.4	3G12620
Hydrolase, TatD family protein, putative	Unique to Zn Presence	n/a	5	18.2	4G07010
BYS1 domain protein, putative	Unique to Zn Presence	n/a	3	17.9	5G01990
Isocyanide synthase XanB	Unique to Zn Presence	n/a	76	72.9	5G02660
Short chain dehydrogenase/reductase family oxidoreductase, putative	Unique to Zn Presence	n/a	16	46	5G09290
Phosphatidylserine decarboxylase family protein	Unique to Zn Presence	n/a	13	60.7	6G00260
40S ribosomal protein S30	Unique to Zn Presence	n/a	5	21	6G02450
O-methyltransferase GliM	Unique to Zn Presence	n/a	28	76.1	6G09680
Ran-interacting protein Mog1, putative	Unique to Zn Presence	n/a	8	69.7	6G12430

Uroporphyrinogen-III synthase (UroS), putative	Unique to Zn Presence	n/a	3	10	6G12800
Metallo-beta-lactamase domain protein, putative	Unique to Zn Presence	n/a	5	14.9	7G00340
Oxidoreductase, 2OG-Fe(II) oxygenase family, putative	Unique to Zn Presence	n/a	6	35.3	8G00110
Alanyl-tRNA synthetase, putative	Unique to Zn Presence	n/a	4	21.6	8G07110
L-PSP endoribonuclease family protein, putative	4.629	0.039406	11	89.3	2G17470
S-(hydroxymethyl)glutathione dehydrogenase	4.473	0.006225	28	60.2	2G01040
Hsp70 chaperone BiP/Kar2, putative	4.362	0.006103	47	57.9	2G04620
Histone H1	3.957	0.015489	15	43.7	3G06070
Cysteine-rich secreted protein	3.937	0.000359	33	79.9	7G01060
Uncharacterised; Upregulated in conidia exposed to neutrophils	3.803	0.003756	8	52.1	1G09030
Glutathione peroxidase	3.730	0.03387	23	46.4	3G12270
Aromatic aminotransferase Aro8, putative	3.724	0.002383	49	87.6	2G13630
L-PSP endoribonuclease family protein (Hmf1), putative	3.569	0.003094	11	60.1	7G02340
Flavin-binding monooxygenase-like protein	2.943	0.004974	45	80.8	4G09220
Aldehyde dehydrogenase, putative	2.927	0.003724	16	51.7	4G02830
Inhibitor I9 domain-containing protein	2.879	0.005395	6	98.6	2G13590
Glycogen synthase kinase (Skp1), putative	2.733	0.010881	13	34	6G05120
Catechol dioxygenase, putative	2.731	0.007808	14	71.4	2G02910
Succinyl-CoA:3-ketoacid-coenzyme A transferase	2.699	0.009881	32	73.2	6G12250
4-aminobutyrate transaminase GatA	2.653	0.00767	50	89.4	5G06680
Isopentenyl-diphosphate delta-isomerase	2.642	0.007262	21	65.9	6G11160
Conserved lysine-rich protein, putative	2.641	0.045629	28	36.4	4G12450
Ortholog of A. nidulans IlmJ – LaeA like methyltransferase	2.617	0.017606	20	72.4	1G10150
GST N-terminal domain-containing protein	2.568	0.002425	17	76.6	3G00730
12-alpha,13-alpha-dihydroxyfumitremorgin C prenyltransferase FtmH	2.544	0.014093	37	74.9	8G00250
Isonitrile hydratase-like protein XanA	2.535	0.038631	11	47.5	5G02670
FG-GAP repeat protein, putative	2.523	0.007388	22	89.9	1G04130
Lactam utilization protein LamB	2.503	0.00929	9	41.5	1G15530
Phospho-2-dehydro-3-deoxyheptonate aldolase	2.494	0.00542	24	76.7	7G04070

Iron/copper transporter Atx1, putative		2.477	0.025248	10	98.7	1G08880
Hydantoin racemase (Dcg1), putative		2.390	0.017439	3	13.6	1G13380
3-hydroxyanthranilate 3,4-dioxygenase 1 (3-HAO-1) Bna1-1		2.367	0.003319	15	98.4	8G04650
Formate dehydrogenase (FDH)		2.302	0.013597	47	66.5	6G04920
Eukaryotic translation initiation factor Eif-5A		2.238	0.018807	14	92.4	1G04070
SAM-dependent methyltransferase GtmA		2.281	0.027096	14	45.6	2G11120
Acetyl-CoA acetyltransferase (erg10B), putative		2.192	0.033505	41	99.2	8G04000
Histidine biosynthesis trifunctional protein		2.133	0.030738	65	81.7	1G14570
Succinate-semialdehyde dehydrogenase, putative		2.073	0.000236	32	84.3	3G07150
Formamidase FmdS		2.062	0.002902	22	55.7	2G02020
Phosphoglycerate mutase, 2,3-bisphosphoglycerate-independent		2.050	0.012541	32	75.4	3G09290
3-isopropylmalate dehydrogenase		2.003	0.014049	16	74.3	1G15780
Peptide hydrolase		1.971	0.000735	15	53	2G00220
Verruculogen synthase FtmF		1.966	0.011072	38	77.3	8G00230
HIT domain protein		1.961	0.01232	15	99.3	6G12680
Cofilin		1.941	0.013675	17	90.3	5G10570
Arylsulfatase, putative		1.931	0.034015	37	50.9	5G12940
Leucine aminopeptidase 2		1.919	0.001467	29	49.5	2G07520
Phosphoglucomutase PgmA		1.918	0.012925	49	96.2	3G11830
Anthranilate synthase component I, putative		1.870	0.014524	16	46.4	6G12580
PH domain protein		1.751	0.021856	31	57.4	6G07910
Phytanoyl-CoA dioxygenase family protein		1.742	0.043023	32	89.5	2G15850
Endothelin-converting enzyme		1.717	0.026816	27	39	3G12420
Antigenic mitochondrial protein HSP60, putative		1.713	0.03311	55	89.4	2G09290
Esterase, putative		1.686	0.048395	25	68	6G02480
3’(2’),5’-bisphosphate nucleotidase		1.658	0.046467	39	62.4	6G09070
Actin cortical patch component, putative		1.653	0.016277	46	81	2G06040
Isocitrate dehydrogenase [NAD] subunit, mitochondrial		1.642	0.003693	19	35.5	6G06370
Diphosphomevalonate decarboxylase		1.639	0.04921	30	82.9	4G07130
Phosphoglycerate kinase		1.627	0.044824	53	97.4	1G10350

Mannitol-1-phosphate 5-dehydrogenase MpdA	1.589	0.045211	66	99.5	2G10660
Dihydroxyacetone kinase (DakA), putative	1.581	0.030092	18	48.6	5G12690
PWWP domain protein	1.553	0.037768	19	44.2	8G04570
Proteasome endopeptidase complex	1.545	0.046827	20	86.9	5G02150
Amine oxidase	1.522	0.018757	46	69.7	5G07360
Extracellular serine-rich protein, putative	1.510	0.019703	15	31.9	3G07870
UBX domain protein, putative	1.505	0.030092	18	40.1	4G07430
Pyruvate decarboxylase, putative	1.490	0.044209	22	60.3	6G00750
6-phosphogluconolactonase (6PGL)	1.476	0.042237	23	99.6	1G02980
Dimeric dihydrodiol dehydrogenase, putative	1.457	0.006893	16	58.3	1G07520
NAD(P)-bd_dom domain-containing protein	1.440	0.009242	21	70.3	2G10170
Peroxiredoxin	1.420	0.037381	20	62.4	5G01440
Beta-lactamase family protein	1.392	0.004739	20	48.6	5G07500
Acylphosphatase-like domain-containing protein	1.374	0.047641	8	53.6	2G05050
Probable glycosidase Crf2	1.344	0.037381	9	17.2	2G03120
Isochorismatase family hydrolase, putative	1.317	0.002066	10	56.7	6G12220
3-oxoacyl-(Acyl-carrier-protein) reductase, putative	1.300	0.027918	16	52.3	3G10540
Vacuolar proton pump subunit B	1.286	0.018077	38	81	2G13240
Oxidoreductase, short chain dehydrogenase/reductase family	1.248	0.033688	13	65.3	5G11240
Amine oxidase	1.223	0.014688	57	82.2	7G04180
Cobalamin-independent methionine synthase MetH/D	1.213	0.010432	83	83.7	4G07360
Transketolase	1.152	0.011109	66	80.8	1G13500
Glycerol dehydrogenase (GldB), putative	1.143	0.008272	37	90.5	4G11730
Probable mannosyl-oligosaccharide alpha-1,2-mannosidase 1B MsdS	1.138	0.023396	21	44.9	1G14560
Proteasome subunit beta	1.075	0.037208	7	29.9	7G04650
Fumarylacetoacetate hydrolase family protein	1.053	0.023208	10	36.2	3G08140
GMC oxidoreductase, putative	1.033	0.041493	32	51.5	3G01580
Lactoylglutathione lyase	1.008	0.018076	25	79.9	6G07940
Agmatinase, putative	1.007	0.032965	18	56.3	5G13180

Supplementary Table S2: Proteins with decreased abundance in *A. fumigatus* wild-type strains grown in Czapek-Dox media with zinc compared to without zinc. Data sorted by fold change, in descending order.

Protein Description	Log2-fold Decrease	P-value	No. of peptides	Sequence coverage (%)	Protein IDs AFUA_
Fatty-acyl coenzyme A oxidase (Pox1), putative	-6.751	0.001207	30	51	7G06090
Major allergen Aspf2	-5.169	0.000117	24	58.3	4G09580
Oxidoreductase, zinc-binding dehydrogenase family, putative	-4.133	0.000224	22	78.8	1G15610
NADH:flavin oxidoreductase/NADH oxidase family protein	-4.104	0.013161	17	56	2G04060
Carboxypeptidase	-3.795	0.038283	6	16.7	2G03510
Proteasome endopeptidase complex	-3.601	0.001201	24	91.3	3G11300
Gliotoxin sulfhydryl oxidase GliT	-3.467	0.005664	38	94.9	6G09740
Pyridoxine biosynthesis protein	-3.450	0.047116	25	77.3	5G08090
12-oxophytodienoate reductase, putative	-3.390	0.002091	14	46.7	5G14330
Thioredoxin reductase	-3.273	0.000805	21	74	4G12990
D-3-phosphoglycerate dehydrogenase	-3.113	0.033926	18	64.7	2G04490
Glutaminase, putative	-3.067	0.000925	18	28.9	3G10910
Myo-inositol-phosphate synthase, putative	-2.985	0.013475	21	39.1	2G01010
Nucleoside hydrolase, putative	-2.905	0.005632	21	41.6	1G11790
NADH-ubiquinone oxidoreductase, subunit G, putative	-2.868	0.020243	33	58.4	5G04370
Alpha-ketoglutarate dehydrogenase complex subunit Kgd1, putative	-2.850	0.017595	61	58	4G11650
1-aminocyclopropane-1-carboxylate deaminase, putative	-2.849	0.003878	34	85.7	2G01030
NADH-dependent flavin oxidoreductase, putative	-2.729	0.003076	21	53.8	7G06420
Adenylosuccinate lyase (Adenylosuccinase)	-2.716	0.003812	30	56.5	2G11940
40S ribosomal protein S3, putative	-2.715	0.015231	26	82.7	1G05630
Catalase B CatB/1	-2.663	0.000198	98	76.8	3G02270
Hsp70 chaperone Hsp88	-2.635	0.006396	65	71.6	1G12610

Proteasome endopeptidase complex		-2.603	0.00611	24	73.2	6G06350
Uricase (Urate oxidase)		-2.542	0.020717	20	62.6	2G10520
Carbamoyl-phosphate synthase, large subunit		-2.497	0.004895	27	28.7	2G10070
Arp2/3 complex subunit (Arp3), putative		-2.491	0.026399	31	82	5G11560
Oxidoreductase, short-chain dehydrogenase/reductase family		-2.403	0.003316	7	34.3	4G03680
Oxidoreductase, 2-nitropropane dioxygenase family, putative		-2.391	0.002726	29	89	2G09850
Cellular morphogenesis protein (Rax2), putative		-2.201	0.028157	11	11.5	7G05340
2-nitropropane dioxygenase family oxidoreductase, putative		-2.146	0.002726	18	59.5	5G09600
Carnitine acetyl transferase		-2.071	0.000878	55	60.4	2G12530
40S ribosomal protein S21		-1.984	0.008378	12	89.8	2G03590
Protein disulphide-isomerase		-1.983	0.038736	39	73.3	2G06150
Homocysteine synthase CysD		-1.974	0.011323	22	56.2	5G04250
SsDNA binding protein, putative		-1.926	0.003918	15	72.5	5G07890
Polyadenylate-binding protein, cytoplasmic and nuclear PAB1		-1.923	0.035214	31	41.3	1G04190
Telomere and ribosome associated protein Stm1, putative		-1.868	0.003513	17	50.3	3G10920
Formyltetrahydrofolate deformylase, putative		-1.863	0.034262	11	58.2	6G11620
Proteasome endopeptidase complex		-1.862	0.046827	20	86.9	5G02150
Cytidine deaminase		-1.855	0.008481	20	82.1	8G02770
Eukaryotic translation initiation factor 2A (Eif-2A)		-1.850	0.002581	9	18.7	3G05970
Proteasome subunit alpha type		-1.844	0.021728	20	64.2	7G05870
3,4-dihydroxy-2-butanone 4-phosphate synthase (DHBP synthase)		-1.838	0.005153	12	59.5	6G13140
60S ribosomal protein L8, putative		-1.823	0.048488	21	78.3	5G06360
Cystathionine gamma-lyase		-1.814	0.011548	8	33.3	8G04340
Adenylosuccinate synthetase (AMPSase)		-1.722	0.007153	24	49.8	1G15450
Alpha-aminoadipate reductase large subunit, putative		-1.709	0.013273	28	27.6	4G11240
Translationally-controlled tumor protein homolog TCTP		-1.702	0.007905	20	90.5	1G16840
S-methyl-5’-thioadenosine phosphorylase (MTAPase)		-1.642	0.006375	20	64.3	6G08720
DNA damage-inducible protein 1 Ddi1		-1.638	0.02637	18	69.6	7G06050
Eukaryotic translation initiation factor 6 (Eif-6)		-1.621	0.001497	10	78.1	5G06010

3-methylcrotonyl-CoA carboxylase, beta subunit (MccB), putative	-1.616	0.015711	21	48.4	5G08940
Carboxypeptidase	-1.601	0.035166	8	23.3	5G07330
Hsp70 chaperone (HscA), putative	-1.567	0.00981	67	84.2	8G03930
Acyl-CoA dehydrogenase family protein	-1.541	0.024196	54	88.1	5G06500
Oxysterol binding protein (Osh5), putative	-1.507	0.002175	12	28.1	3G11750
Glycine cleavage system H protein	-1.457	0.002743	13	46.3	1G12070
Pyruvate dehydrogenase E1 component subunit beta	-1.443	0.007359	22	63.4	3G04170
Pyruvate dehydrogenase E1 component subunit alpha	-1.432	0.008532	45	87.6	1G06960
Rab GDP dissociation inhibitor	-1.417	0.036762	38	83.8	2G11150
Peroxiredoxin Asp f3 (Prx) (Aspf3)	-1.411	0.005267	31	99.4	6G02280
Enoyl-CoA hydratase/isomerase family protein	-1.387	0.046823	13	51.4	2G14850
D-3-phosphoglycerate dehydrogenase	-1.318	0.014822	22	69.8	5G05500
Kynurenine aminotransferase, putative	-1.297	0.015787	12	24.4	4G11190
Argininosuccinate synthase	-1.283	0.014827	28	83.9	2G04310
Nascent polypeptide-associated complex subunit beta (NAC-beta) Egd1	-1.280	0.005629	9	71	6G02750
FK506-binding protein 4 (Histone proline isomerase (Rotamase) Fpr4	-1.268	0.018187	11	28.8	6G08580
Ser/Thr protein phosphatase family	-1.227	0.048207	14	37.9	3G04160
Pyridoxamine phosphate oxidase, putative	-1.190	0.025172	9	67.9	3G06670
Acyl-CoA thioesterase II	-1.157	0.000793	7	31.5	1G15170
Heat shock protein 90 (Heat shock protein hsp1) (Aspf12)	-1.132	0.017218	63	68.1	5G04170
Peroxisomal multifunctional beta-oxidation protein (MFP), putative	-1.084	0.002293	70	75.9	4G03900
Proline iminopeptidase	-1.051	0.010567	12	35.6	2G05000
Mannitol 2-dehydrogenase M2DH	-1.049	0.035599	16	32.3	4G14450
Kelch repeat protein	-1.041	0.01224	30	83.7	5G12780
Alkaline protease 2 (ALP2) (Aspf18)	-1.021	0.029356	29	42.8	5G09210
N-acetyltransferase family protein, putative	-1.000	0.008915	16	58.4	4G10930
Coatomer subunit delta	Unique to Zn Absence	n/a	13	25.2	1G15860

Dihydrodipicolinate synthetase family protein	Unique to Zn Absence	n/a	9	33.5	2G01230
Putative tyrosine decarboxylase, Transcript induced by voriconazole	Unique to Zn Absence	n/a	12	35	2G04980
Kynureninase 1	Unique to Zn Absence	n/a	9	31.4	2G10360
40S ribosomal protein S27	Unique to Zn Absence	n/a	5	43.9	3G06640
Alpha-mannosidase	Unique to Zn Absence	n/a	53	45.9	3G08200
Coatomer subunit alpha	Unique to Zn Absence	n/a	18	16.9	3G08840
Putatative Mitochondrial cytochrome b2	Unique to Zn Absence	n/a	17	48.8	4G03120
Glutamyl-tRNA(Gln) amidotransferase, subunit A	Unique to Zn Absence	n/a	12	65.8	5G07300
Isoflavone reductase family protein	Unique to Zn Absence	n/a	11	41.7	5G09450
L-amino acid oxidase LaoA	Unique to Zn Absence	n/a	22	40.5	7G06810

Supplementary Table S3: Proteins with increased abundance (or unique) in *A. fumigatus ΔgtmA* strains grown in Czapek-Dox media with zinc compared to without zinc. Data sorted by fold change, in descending order.

Protein Description	Log2-fold Increase	P-value	No. of peptides	Sequence coverage (%)	Protein IDs AFUA_
N-acetylglucosamine-6-phosphate deacetylase	Unique to Zn Presence	n/a	5	20.7	1G00450
T-complex protein 1, beta subunit, putative	Unique to Zn Presence	n/a	13	31.8	1G01740
Peptidyl-prolyl cis-trans isomerase, putative	Unique to Zn Presence	n/a	7	15	1G01750
GYF domain-containing protein	Unique to Zn Presence	n/a	5	13.2	1G02420
Glycosyl hydrolase, putative	Unique to Zn Presence	n/a	9	19.7	1G03140
HMG box protein, putative	Unique to Zn Presence	n/a	6	21	1G04550
Dynein light chain	Unique to Zn Presence	n/a	4	48.9	1G04850
Protein phosphatase	Unique to Zn Presence	n/a	4	7.2	1G05640
6-phosphofructo-2-kinase 1	Unique to Zn Presence	n/a	7	20.9	1G07220
Myosin class II heavy chain (MHC), putative	Unique to Zn Presence	n/a	4	2.8	1G11450
Catechol dioxygenase, putative	Unique to Zn Presence	n/a	3	9.7	1G14270
tRNAHis guanylyltransferase Thg1, putative	Unique to Zn Presence	n/a	5	15.4	1G14630
Acyl-CoA thioesterase II	Unique to Zn Presence	n/a	7	31.5	1G15170
Polysaccharide deacetylase family protein	Unique to Zn Presence	n/a	2	8	1G15280
Ubiquitin carboxyl-terminal hydrolase	Unique to Zn Presence	n/a	5	16.8	1G15920
Probable beta-galactosidase B LacB	Unique to Zn Presence	n/a	6	7.3	1G16700
Beta-N-hexosaminidase, putative	Unique to Zn Presence	n/a	5	10.2	2G00640
FAD binding domain protein	Unique to Zn Presence	n/a	5	14.9	2G00730
GNAT family N-acetyltransferase, putative	Unique to Zn Presence	n/a	9	60.2	2G01380
DUF21 and CBS domain protein (Mam3), putative	Unique to Zn Presence	n/a	3	5.9	2G04430
RNA binding effector protein Scp160, putative	Unique to Zn Presence	n/a	11	9.7	2G04700
Cell pattern formation-associated protein StuA	Unique to Zn Presence	n/a	3	7.1	2G07900
Eukaryotic translation initiation factor subunit Eif-4F, putative	Unique to Zn Presence	n/a	7	10.2	2G09490

Protein arginine methyltransferase NDUFAF7	Unique to Zn Presence	n/a	6	15.5	2G09740
Inositol oxygenase, putative	Unique to Zn Presence	n/a	3	19.4	2G10230
NAD binding Rossmann fold oxidoreductase, putative	Unique to Zn Presence	n/a	7	25.6	2G10240
Actin-related protein 8	Unique to Zn Presence	n/a	4	9.2	2G10990
Acyl CoA binding protein family	Unique to Zn Presence	n/a	7	72.2	2G11060
CoA_binding domain-containing protein	Unique to Zn Presence	n/a	2	20.3	2G11650
Acetylornithine aminotransferase	Unique to Zn Presence	n/a	8	27.9	2G12470
Oxidoreductase, FAD-binding, putative	Unique to Zn Presence	n/a	3	8.2	2G14480
Oxalate decarboxylase, putative	Unique to Zn Presence	n/a	5	16.3	2G14610
Lectin family integral membrane protein, putative	Unique to Zn Presence	n/a	5	12.9	2G16800
Alpha/beta hydrolase, putative	Unique to Zn Presence	n/a	5	24.7	3G01280
NUDIX domain, putative	Unique to Zn Presence	n/a	2	12.9	3G02870
Ubiquitin conjugating enzyme (UbcD), putative	Unique to Zn Presence	n/a	4	45.6	3G06030
Metallo-beta-lactamase superfamily protein	Unique to Zn Presence	n/a	5	49.2	3G07630
Extracellular thaumatin domain protein, putative	Unique to Zn Presence	n/a	3	30.5	3G09690
3-hydroxy-3-methylglutaryl coenzyme A synthase (HMG-CoA synthase)	Unique to Zn Presence	n/a	6	30.7	3G10660
Methyltransf_11 domain-containing protein	Unique to Zn Presence	n/a	2	11.2	3G10710
NAD binding Rossmann fold oxidoreductase, putative	Unique to Zn Presence	n/a	4	15	3G11930
pH-response transcription factor PacC	Unique to Zn Presence	n/a	4	6.4	3G11970
Stomatin family protein	Unique to Zn Presence	n/a	8	30	3G13440
Glycolipid transfer protein HET-C2, putative	Unique to Zn Presence	n/a	6	47.8	3G13820
Enoyl-CoA hydratase/isomerase family protein	Unique to Zn Presence	n/a	8	42.8	3G14520
Acid phosphatase, putative	Unique to Zn Presence	n/a	2	15	4G01070
N,N-dimethylglycine oxidase	Unique to Zn Presence	n/a	6	24.8	4G01150
Acetyltransferase, GNAT family, putative	Unique to Zn Presence	n/a	4	35.3	4G02980
Cell wall serine-threonine-rich galactomannoprotein Mp1	Unique to Zn Presence	n/a	3	17.3	4G03240
Oxidoreductase, short-chain dehydrogenase/reductase family	Unique to Zn Presence	n/a	7	34.3	4G03680
Copper resistance protein Crd2, putative	Unique to Zn Presence	n/a	3	30.6	4G04318
PaaI_thioesterase family protein, putative	Unique to Zn Presence	n/a	4	17.7	4G04355

Allergen Aspf7	Unique to Zn Presence	n/a	3	15.6	4G06670
Integral ER membrane protein Scs2, putative	Unique to Zn Presence	n/a	4	27.8	4G06950
Aspartic-type endopeptidase ctsD	Unique to Zn Presence	n/a	5	13.9	4G07040
Glycylpeptide N-tetradecanoyltransferase Nmt1	Unique to Zn Presence	n/a	9	23	4G08070
Dihydrodipicolinate synthetase family protein	Unique to Zn Presence	n/a	9	37.8	4G08690
Phosphotransmitter protein Ypd1, putative	Unique to Zn Presence	n/a	3	20.5	4G10280
Homocitrate synthase	Unique to Zn Presence	n/a	7	21	4G10460
Trehalase (Alpha-trehalose glucohydrolase)	Unique to Zn Presence	n/a	6	5.8	4G13530
Transesterase (LovD), putative	Unique to Zn Presence	n/a	2	12	5G00920
UPF0047 domain protein	Unique to Zn Presence	n/a	6	53.5	5G02090
Queuine Trna-ribosyltransferase accessory subunit 2	Unique to Zn Presence	n/a	8	22.5	5G03470
Endochitinase A1 ChiA1	Unique to Zn Presence	n/a	3	4.2	5G03760
RNA binding protein Nrd1, putative	Unique to Zn Presence	n/a	3	5.2	5G04240
Pre-mRNA-splicing factor Cwc2	Unique to Zn Presence	n/a	2	6	5G06760
Nuclear migration protein (ApsA), putative	Unique to Zn Presence	n/a	6	4.3	5G07710
Transcription factor RfeG, putative	Unique to Zn Presence	n/a	4	26.1	5G08990
Phosphatidylinositol transporter, putative	Unique to Zn Presence	n/a	4	10.2	5G09260
FHA domain protein	Unique to Zn Presence	n/a	3	6.4	5G13560
Beta-galactosidase, putative	Unique to Zn Presence	n/a	5	9.6	5G14550
DUF453 domain protein	Unique to Zn Presence	n/a	6	27.1	6G00360
Probable arabinan endo-1,5-alpha-L-arabinosidase C ABNC	Unique to Zn Presence	n/a	5	28.4	6G00770
Conidiation-specific protein (Con-10), putative	Unique to Zn Presence	n/a	2	47	6G03210
Protein kinase domain-containing protein	Unique to Zn Presence	n/a	3	11.7	6G03240
Mannosyl-oligosaccharide glucosidase, putative	Unique to Zn Presence	n/a	10	19.3	6G04210
RNA polymerase II Elongator subunit, putative	Unique to Zn Presence	n/a	4	6.6	6G05090
Short chain dehydrogenase, putative	Unique to Zn Presence	n/a	5	40.5	6G06930
Stromal membrane-associated protein	Unique to Zn Presence	n/a	6	16.4	6G07830
Probable catabolite repression protein CreC	Unique to Zn Presence	n/a	4	7	6G07860
CwfJ domain protein	Unique to Zn Presence	n/a	5	13.3	6G08250
Zinc metallopeptidase, putative	Unique to Zn Presence	n/a	7	26.6	6G09600

Glutaminase, putative	Unique to Zn Presence	n/a	11	17.8	6G09910
Patched sphingolipid transporter (Ncr1), putative	Unique to Zn Presence	n/a	7	10.2	6G09980
D-isomer specific 2-hydroxyacid dehydrogenase family protein	Unique to Zn Presence	n/a	9	32.9	6G10090
Possible replication factor-a protein	Unique to Zn Presence	n/a	2	11.8	6G11130
ABC transporter, putative	Unique to Zn Presence	n/a	4	9	6G11360
Dynamin GTPase, putative	Unique to Zn Presence	n/a	6	8.9	6G11890
Glycerophosphoryl diester phosphodiesterase family protein	Unique to Zn Presence	n/a	10	23.9	6G12240
CRAL/TRIO domain protein	Unique to Zn Presence	n/a	4	9	6G12690
26S proteasome non-ATPase regulatory subunit Nas2, putative	Unique to Zn Presence	n/a	5	31	6G13310
UPF0023 family protein	Unique to Zn Presence	n/a	3	7.3	6G13530
DnaJ domain protein	Unique to Zn Presence	n/a	3	7.7	7G01230
RPEL repeat protein	Unique to Zn Presence	n/a	3	35.9	7G01340
RhoGAP and Fes/CIP4 domain protein	Unique to Zn Presence	n/a	7	15	7G04300
Gamma-glutamyltranspeptidase	Unique to Zn Presence	n/a	8	18.7	7G04760
Cellular morphogenesis protein (Rax2), putative	Unique to Zn Presence	n/a	11	11.5	7G05340
Glucanase, putative	Unique to Zn Presence	n/a	4	14.3	7G05610
Threonine aldolase, putative	Unique to Zn Presence	n/a	2	5.6	7G06540
L-amino acid oxidase LaoA	Unique to Zn Presence	n/a	22	40.5	7G06810
Endo-chitosanase	Unique to Zn Presence	n/a	5	30.6	8G00930
Vacuolar protein sorting/targeting protein 10 Vsp10	Unique to Zn Presence	n/a	7	7.1	8G02780
Peptidyl-prolyl cis-trans isomerase H Cyp3	Unique to Zn Presence	n/a	4	48.7	8G03890
Glucosamine-6-phosphate isomerase	Unique to Zn Presence	n/a	6	14.6	8G04070
Flavohemoprotein, putative	Unique to Zn Presence	n/a	2	8.3	8G06080
Pyruvate carboxylase	3.557	0.006854	73	69.7	4G07710
Glycerol kinase, putative	3.490	0.002197	26	50.9	6G08470
Probable beta-glucosidase A BglA	3.210	0.000732	29	33.5	1G05770
Major allergen Aspf2	3.208	0.006288	24	58.3	4G09580
Peptidyl-arginine deiminase domain protein	3.181	0.006223	36	79.4	8G06520
6-hydroxytryprostatin B O-methyltransferase FtmD	2.993	0.001055	55	87.6	8G00200
Glutamine synthetase	2.742	0.000171	32	70.1	4G13120

Glycogen debranching enzyme Gdb1, putative	2.707	0.011795	30	29.4	1G02140
Mannitol-1-phosphate 5-dehydrogenase MpdA	2.694	0.001817	66	99.5	2G10660
Catalase B CatB/1	2.432	0.002146	98	76.8	3G02270
Superoxide dismutase [Mn], mitochondrial (allergen Aspf6) SodC	2.429	0.037797	31	97.1	1G14550
Glutamate synthase Glt1, putative	2.369	0.003505	27	15.6	1G07380
PT repeat family protein	2.329	0.034549	9	8.2	2G17000
Thioredoxin, putative	2.327	0.020949	11	97.2	8G01090
Glutathione peroxidase	2.322	0.039977	23	46.4	3G12270
Methionine aminopeptidase 2-2 (MAP 2-2)	2.257	0.022667	28	70.7	2G01750
Histidine biosynthesis trifunctional protein	2.250	0.001919	65	81.7	1G14570
Aspartic-type endopeptidase, putative	2.179	0.000387	9	22.4	4G09400
Tripeptidyl-peptidase Sed2	2.154	0.044029	17	31.4	4G03490
Short chain dehydrogenase, putative	2.118	0.000899	30	75.3	4G08710
Urease Ure1	2.102	0.002751	25	47.5	1G04560
Iron/copper transporter Atx1, putative	2.083	0.043061	10	98.7	1G08880
Methylthioribose-1-phosphate isomerase Mri1	2.065	0.001905	16	65.1	4G05830
2-amino-3-carboxymuconate-6-semialdehyde decarboxylase, putative	2.019	0.044251	11	39.4	5G12460
Probable mannosyl-oligosaccharide alpha-1,2-mannosidase 1B MsdS	1.998	0.024429	21	44.9	1G14560
Oxidoreductase, short chain dehydrogenase/reductase family	1.984	0.008346	10	36.5	6G13830
Phosphatidylglycerol/phosphatidylinositol transfer protein (PG/PI-TP) Npc2	1.939	0.006881	14	35.5	2G11340
Translation machinery-associated protein 20	1.934	0.010024	9	52.7	1G09810
Aldose 1-epimerase, putative	1.933	0.030313	17	60.1	3G05740
Epoxide hydrolase, putative	1.808	0.002641	18	42.2	5G08810
1-aminocyclopropane-1-carboxylate deaminase, putative	1.765	0.000393	34	85.7	2G01030
Homoserine dehydrogenase (HDH)	1.760	0.048981	21	76.4	3G11640
Probable NAD(P)H-dependent D-xylose reductase Xyl1	1.755	0.015291	24	62.9	1G04820
Probable Xaa-Pro aminopeptidase	1.736	0.011834	18	49	1G14920
Hsp90 binding co-chaperone (Sba1), putative	1.736	0.038879	13	75.1	5G13920
Fumarylacetoacetate hydrolase family protein	1.723	0.000725	24	59.8	6G06460

Orotate phosphoribosyltransferase	1.718	0.033269	22	87.4	2G11290
1,3-beta-glucanosyltransferase Bgt1	1.649	0.033057	9	30.8	1G11460
DUF636 domain protein	1.641	0.001002	13	91.9	2G15290
Alcohol dehydrogenase, zinc-containing, putative	1.625	0.046457	34	90.8	1G04620
Flavin-binding monooxygenase-like protein	1.623	0.025924	45	80.8	4G09220
Gliotoxin sulfhydryl oxidase GliT	1.619	0.002884	38	94.9	6G09740
1,3-beta-glucanosyltransferase Gel1	1.601	0.026083	26	61.5	2G01170
Triosephosphate isomerase	1.590	0.049718	35	91.2	5G13450
Pyruvate kinase	1.580	0.008909	51	92.6	6G07430
NAD binding Rossmann fold oxidoreductase, putative	1.571	0.027833	13	32	3G11840
Oxidoreductase, 2OG-Fe(II) oxygenase family, putative	1.530	0.012105	17	56.6	3G00800
Isochorismatase family hydrolase, putative	1.496	0.024206	3	24.7	2G03770
12-alpha,13-alpha-dihydroxyfumitremorgin C prenyltransferase FtmH	1.484	0.015104	37	74.9	8G00250
Superoxide dismutase	1.482	0.033777	14	72.9	4G11580
HAD superfamily hydrolase, putative	1.469	0.000649	13	65.6	5G08270
UTP--glucose-1-phosphate uridylyltransferase	1.433	0.026378	52	88.7	7G01830
Inosine-5'-monophosphate dehydrogenase	1.429	0.001766	24	50.5	2G03610
Probable glycosidase Crf2	1.427	0.007884	9	17.2	2G03120
Asp-hemolysin (Asp-HS)	1.393	0.000845	24	74.8	3G00590
FAD-dependent oxygenase, putative	1.368	0.009076	25	45.4	3G00840
Methionine aminopeptidase 2-1 (MAP 2-1)	1.360	0.019253	24	41	8G00410
Isocitrate lyase (Icl1) (Isocitrase)	1.347	0.000496	37	72.3	4G13510
Catalase A CatA	1.337	0.01214	72	86.1	6G03890
Sterol carrier protein, putative	1.331	0.036314	24	56.8	4G06380
Dienelactone hydrolase family protein	1.322	0.049382	21	61	6G01940
Oxidoreductase, short-chain dehydrogenase/reductase family, putative	1.311	0.040876	10	47.7	5G14340
Peptide hydrolase	1.309	0.000381	15	53	2G00220
Molybdopterin molybdenumtransferase	1.288	0.020014	12	33	2G15550
Trimethyllysine dioxygenase TmlH, putative	1.281	0.042083	16	29.8	1G06180

Isopentenyl-diphosphate delta-isomerase	1.281	0.005804	21	65.9	6G11160
Nucleoside diphosphate kinase Ndk1	1.279	0.011192	23	100	5G03490
Rho-gdp dissociation inhibitor	1.274	0.0039	14	73.1	5G11380
Cdc48-dependent protein degradation adaptor protein (Shp1), putative	1.271	0.04207	16	43.1	5G03610
Acid phosphatase, putative	1.261	0.038382	46	66.7	1G16480
Ubiquitin conjugating enzyme (UbcA), putative	1.242	0.024058	9	65.1	5G07040
Phosphoribosylglycinamide formyltransferase, putative	1.242	0.022935	13	71	2G10855
Actin-related protein 2/3 complex subunit	1.224	0.035185	23	72.2	6G06500
Xanthine dehydrogenase HxA, putative	1.221	0.026852	26	31.4	4G11220
Fe2OG dioxygenase domain-containing protein	1.220	0.022522	11	41.6	1G04760
Cell wall biogenesis protein phosphatase Ssd1, putative	1.217	0.000644	39	39	1G11420
Vacuolar aspartyl aminopeptidase Lap4, putative	1.215	0.041329	41	66.9	5G03990
Ras GTPase Rab11, putative	1.214	0.01818	12	40.5	1G02190
Succinate--CoA ligase [ADP-forming] subunit beta, mitochondrial	1.212	0.011043	36	69.6	4G04520
Calcium homeostasis protein Regucalcin, putative	1.211	0.037047	20	80.4	4G08610
G2/M phase checkpoint control protein Sum2, putative	1.186	0.008851	8	18.5	1G12390
3-ketoacyl-coA thiolase peroxisomal A	1.182	0.040628	41	84	4G10950
Glycogen synthase kinase (Skp1), putative	1.175	0.028141	13	34	6G05120
Probable glucan endo-1,3-beta-glucosidase EglC	1.164	0.015421	23	33.2	3G00270
Mandelate racemase/muconate lactonizing enzyme family protein	1.150	0.003928	13	55.4	1G05520
Lipid transfer protein, putative	1.141	0.00488	5	55.8	6G13280
Oxidoreductase, zinc-binding dehydrogenase family, putative	1.129	0.040982	22	78.8	1G15610
Pyruvate dehydrogenase E1 component subunit beta	1.113	0.003901	22	63.4	3G04170
Peroxisomal dehydratase, putative	1.108	0.005565	8	50.3	5G00640
Obg-like ATPase 1	1.094	0.042302	31	86.5	1G09800
Beta-glucosidase, putative	1.089	0.000485	13	42.2	1G14710
Vacuolar protease A (Aspartic endopeptidase Pep2)	1.083	0.012788	22	49.5	3G11400
HIT domain protein	1.083	0.001794	15	99.3	6G12680
Endothelin-converting enzyme	1.081	0.020787	27	39	3G12420

Probable alpha-galactosidase B AglB	1.077	0.019537	10	28.6	5G02130
Thioredoxin	1.066	0.030173	22	99.1	5G11320
Ribose 5-phosphate isomerase A	1.062	0.029038	18	75.7	6G10610
Peroxisomal multifunctional beta-oxidation protein (MFP), putative	1.056	0.014895	70	75.9	4G03900
Coatomer subunit epsilon	1.056	0.001549	10	62.8	1G13260
RAB GTPase Vps21/Ypt51, putative	1.050	0.025869	18	69.1	3G10740
Tryprostatin B synthase FtmB	1.048	0.046751	27	69.8	8G00210
MHD domain-containing protein	1.035	0.006253	12	21	1G13590
DUF833 domain protein	1.033	0.010788	17	56.5	2G09530
Pyridox_oxase_2 domain-containing protein	1.023	0.025634	18	65.4	5G10990
Cytochrome b5, putative	1.022	0.039445	7	23	2G04710
2-nitropropane dioxygenase family oxidoreductase, putative	1.018	0.012	18	59.5	5G09600
Mitogen-activated protein kinase	1.009	0.022023	22	49.1	4G13720
Glycosidase	1.004	0.04032	8	27.8	6G08510
Short chain dehydrogenase/reductase family protein	1.000	0.002893	21	66.4	4G14010

Supplementary Table S4: Proteins with decreased abundance in *A. fumigatus ΔgtmA* strains grown in Czapek-Dox media with zinc compared to without zinc. Data sorted by fold change, in descending order.

Protein Description	Log2-fold Decrease	P-value	No. of peptides	Sequence coverage (%)	Protein IDs AFUA_
Amino acid oxidase FmpA	-7.853	0.000558	43	85.5	6G03440
Fumipyrrole biosynthesis protein C FmpC	-6.311	0.006005	17	33.3	6G03460
Heat shock protein Hsp30/Hsp42, putative	-6.086	0.001133	19	85.6	3G14540
60S ribosomal protein L3 (allergen Aspf23)	-5.751	0.006971	23	55.4	2G11850
PKS-NRPS hybrid synthetase PsoA	-5.524	0.000525	112	42.8	8G00540
Phenol 2-monooxygenase, putative	-4.884	0.002782	35	58	1G13660
Flavin-containing monooxygenase, putative	-4.854	0.000926	46	80.5	5G00310
Aminotransferase, classes I and II, putative	-4.712	0.006537	12	43.3	4G11460
Mitochondrial ADP,ATP carrier protein (Ant), putative	-4.343	0.011562	21	48.1	1G05390
Isocyanide synthase XanB	-4.105	0.03158	76	72.9	5G02660
40S ribosomal protein S3, putative	-3.970	0.002234	26	82.7	1G05630
FAD-dependent oxidase, putative	-3.898	0.000475	10	15.7	1G00980
60S ribosomal protein L11	-3.868	0.010598	11	51.7	4G07730
40S ribosomal protein S15, putative	-3.771	0.018847	6	28.8	2G10090
Eukaryotic translation initiation factor 3 subunit D (eIF3d)	-3.768	0.047152	20	55.4	2G14670
40S ribosomal protein S4	-3.767	0.02365	21	71.3	3G06840
60S ribosomal protein L9, putative	-3.627	0.044071	11	60.4	1G09100
O-methyltransferase GliM	-3.595	0.010986	28	76.1	6G09680
NAD-specific glutamate dehydrogenase	-3.531	0.002253	47	47.8	2G06000
Oxidoreductase, short-chain dehydrogenase/reductase family	-3.481	0.000436	16	73.2	5G09150
Aldehyde reductase (GliO-like), putative	-3.446	0.01467	37	74.7	5G02020
40S ribosomal protein S8	-3.428	0.043406	16	58.2	1G04320
40S ribosomal protein S1	-3.373	0.009138	21	63.7	5G05450
Extracellular metalloproteinase Mep (Allergen Aspf5)	-3.323	0.009946	16	38.5	8G07080

Heat shock protein Hsp98/Hsp104/ClpA, putative	-3.309	0.037895	52	52.8	1G15270
40S ribosomal protein S11	-3.305	0.010453	13	84	2G10440
Serine hydroxymethyltransferase	-3.227	0.004437	47	95.1	3G09320
Proteasome endopeptidase complex	-3.194	0.026253	24	91.3	3G11300
Outer mitochondrial membrane protein porin	-3.168	0.018036	24	95.8	4G06910
60S ribosomal protein L24a	-3.164	0.030331	12	45.6	6G02440
Toxin biosynthesis protein GliH, putative	-3.097	0.000581	20	82.1	6G09745
Thiol methyltransferase, putative	-2.991	0.000371	18	67	4G13570
Amine oxidase	-2.960	0.004813	45	82.3	3G14590
Cell division control protein Cdc48	-2.943	0.000697	74	85	2G17110
Ubiquitin C-terminal hydrolase (HAUSP), putative	-2.929	0.021582	25	32.4	5G03250
Allergen Aspf1	-2.893	0.000655	10	67	5G02330
Cyclopropane-fatty-acyl-phospholipid synthase, putative	-2.890	0.018173	9	31.2	7G04190
Tubulin beta chain	-2.888	0.004481	27	64.8	1G10910
Proteasome subunit alpha type	-2.870	0.001109	14	90.1	6G04790
Cytochrome P450 monooxygenase GliF	-2.769	0.006688	19	32.7	6G09730
Translation elongation factor eEF-1 subunit gamma, putative	-2.762	0.014297	22	52.5	6G04570
O-methyltransferase af390-400 FmaD	-2.748	0.000217	20	83.5	8G00390/400
Zinc-containing alcohol dehydrogenase, putative	-2.724	0.012316	18	52.7	4G08240
Amine oxidase	-2.720	0.011341	38	60.3	3G00680
Lysophospholipase, putative	-2.681	0.031962	15	45.5	5G02930
Alpha mannosidase	-2.679	0.002238	53	45.9	3G08200
Amine oxidase	-2.656	0.016608	57	82.2	7G04180
Proteasome endopeptidase complex	-2.644	0.001238	24	77.2	2G11440
Glutamate-cysteine ligase Gcs1, putative	-2.619	0.014585	14	27.4	3G13900
Tubulin alpha chain	-2.586	0.004103	23	60.9	1G02550
Anthranilate phosphoribosyltransferase, putative	-2.562	0.000917	11	50.6	4G11980
RNA binding protein, putative	-2.545	0.011865	9	98.2	6G13330
ATP synthase subunit gamma	-2.540	0.002369	24	74.4	1G03510
5-oxo-L-prolinase, putative	-2.502	0.045899	34	42.3	6G14330

60S ribosomal protein L8, putative	-2.467	0.042242	21	78.3	5G06360
CipC-like antibiotic response protein, putative	-2.458	0.006169	5	56.9	5G09330
Aryl-alcohol dehydrogenase Aad14, putative	-2.433	0.00536	23	72.7	2G11250
Aldehyde dehydrogenase, putative	-2.422	0.000478	16	51.7	4G02830
Isonitrile hydratase-like protein XanA	-2.410	0.017907	11	47.5	5G02670
C1 tetrahydrofolate synthase, putative	-2.398	0.007755	37	47	3G08650
Polyketide transferase af380 FmaC	-2.395	0.007973	24	93.2	8G00380
Ribosomal protein L26	-2.353	0.024714	11	17.3	6G11260
14-3-3 family protein ArtA, putative	-2.350	0.000378	13	50.6	2G03290
CAIB/BAIF family enzyme	-2.342	0.003098	18	49	3G09240
Actin Act1	-2.317	0.005897	32	70.5	6G04740
N-methyltransferase GliN	-2.309	0.008274	36	99.6	6G09720
40S ribosomal protein S7	-2.308	0.005938	22	68.7	3G10730
60S ribosomal protein L31e	-2.299	0.040496	11	68.3	6G13250
Plasma membrane ATPase	-2.278	0.030018	34	36.8	3G07640
40S ribosomal protein S5, putative	-2.260	0.039495	14	53	1G15020
Pentafunctional AROM polypeptide AroM	-2.254	0.002711	26	22.6	1G13740
Cysteine-rich secreted protein	-2.217	0.031353	33	79.9	7G01060
S-adenosylmethionine synthase	-2.210	0.01735	36	93	1G10630
Ribosomal protein L14	-2.186	0.026109	14	72.3	6G03830
Maleylacetoacetate isomerase MaiA	-2.175	0.000809	14	61	2G04240
ATP-dependent 6-phosphofructokinase PfkA	-2.158	0.036342	33	48.9	4G00960
Aflatoxin B1-aldehyde reductase GliO-like, putative	-2.151	0.000121	11	49.1	1G13370
Histone H3 HhtA	-2.035	0.00543	9	60.3	1G13790
2-oxoisovalerate dehydrogenase subunit alpha	-2.013	0.002095	14	54.7	6G08830
TauD domain-containing protein	-2.001	0.001716	13	40.3	2G05220
60S ribosomal protein L12	-1.990	0.029749	8	46.1	1G03390
Oxidoreductase CipA-like, putative	-1.987	0.00801	16	42.3	1G01630
Dioxygenase af480 FmaF	-1.980	0.001868	7	30.1	8G00480
Phosphoenolpyruvate carboxykinase AcuF	-1.976	0.003126	31	62.9	6G07720

Indoleamine 2,3-dioxygenase family protein		-1.937	0.012032	11	28.4	7G02010
FAD binding domain protein		-1.933	0.044635	23	59	3G12950
Septin		-1.916	0.022657	21	54.4	5G03080
Phosphoketolase, putative		-1.902	0.00275	35	45.2	3G00370
Lipase, putative		-1.897	0.04891	14	44.9	7G04020
ATP-dependent RNA helicase Eif4A		-1.892	0.006437	27	73.6	3G08160
Glycerol dehydrogenase Gcy1, putative		-1.865	0.008692	25	66.2	1G09930
Homocysteine synthase CysD		-1.862	0.000905	22	56.2	5G04250
Eukaryotic translation elongation factor 1 subunit Eef1-beta, putative		-1.854	0.011232	24	92.5	1G11190
EthD domain-containing protein		-1.834	0.029672	19	94.3	8G00430
Glucosamine-fructose-6-phosphate aminotransferase		-1.830	0.036803	23	45.7	6G06340
Fumarate reductase		-1.829	0.000642	43	80.1	7G05070
IgE-binding protein		-1.799	0.020598	2	16	6G00430
YjgH family protein		-1.789	0.000375	12	72.2	3G02253
Ribosomal protein S5		-1.782	0.013677	12	38.2	7G01460
Gamma-glutamyltranspeptidase		-1.773	0.00523	24	63.3	4G13580
Dihydrolipoamide succinyltransferase, putative		-1.762	0.014249	16	38.6	3G05370
Protein transport protein Sec13		-1.733	0.007935	8	39.2	4G06090
Importin subunit alpha		-1.713	0.028855	19	42.4	2G16090
40S ribosomal protein S17, putative		-1.691	0.04955	12	46	2G10300
Beta-lactamase		-1.688	0.018982	13	49.6	5G09790
Cystathionine beta-synthase		-1.686	0.000228	27	67.2	2G07620
Antigenic mitochondrial protein HSP60, putative		-1.682	0.005342	55	89.4	2G09290
Proteasome endopeptidase complex		-1.669	0.002895	24	73.2	6G06350
Probable acetate kinase		-1.666	0.005869	7	31.8	3G10750
Oxidoreductase, short chain dehydrogenase/reductase family		-1.655	0.001757	23	87.8	8G05590
DUF636 domain protein		-1.652	0.024345	11	57.7	1G09754
Dual-functional monooxygenase/ methyltransferase PsoF		-1.652	0.005662	31	43.9	8G00440
Trehalose synthase (Ccg-9), putative		-1.645	0.023323	26	54.5	3G12100
Aminotransferase, putative		-1.639	0.001234	4	13.2	6G00290

Zinc-binding oxidoreductase ToxD, putative	-1.598	0.011449	22	67.1	6G10120
Short chain dehydrogenase/reductase family protein	-1.592	0.026406	14	75.1	1G00990
Acetyl-CoA-acetyltransferase, putative	-1.581	0.000715	32	83.4	6G14200
26S proteasome regulatory particle subunit Rpn8, putative	-1.546	0.030258	19	83.7	1G07540
Nicotinate-nucleotide pyrophosphorylase [carboxylating]	-1.526	0.012507	10	44.1	3G05730
Chorismate synthase	-1.506	0.018738	27	79.6	1G06940
NmrA-like family protein, putative	-1.492	0.014489	27	80.8	4G02840
Phosphoglycerate kinase	-1.464	0.033	53	97.4	1G10350
Prolyl-Trna synthetase	-1.464	0.047026	11	29.1	2G16010
40S ribosomal protein S22	-1.463	0.012531	11	70	1G15730
Aldo-keto reductase (AKR13), puatative	-1.441	0.013056	36	95.6	7G00700
Histone acetyltransferase type B subunit 2 Hat2	-1.431	0.007671	12	53.3	5G03130
Succinate dehydrogenase [ubiquinone] iron-sulfur subunit, mitochondrial	-1.414	0.02287	16	53.2	5G10370
Ctr copper transporter family protein	-1.408	0..046326	6	29.2	2G03730
Citrate synthase	-1.382	0.041732	35	68.6	6G03590
Beta-alanine synthase, putative	-1.381	0.000893	12	33.7	6G12670
Isocitrate dehydrogenase [NAD] subunit, mitochondrial	-1.333	0.007578	19	35.5	6G06370
Methyltransferase PsoC	-1.330	0.002107	42	81.5	8G00550
Hsp70 chaperone (BiP), putative	-1.326	0.034841	12	44.7	2G02320
N-acetyltransferase domain-containing protein	-1.293	0.041163	11	47.1	4G09550
Short-chain dehydrogenase, putative	-1.290	0.017491	19	57.5	8G00280
NAD binding Rossmann fold oxidoreductase, putative	-1.283	0.000513	34	91.4	5G07000
Cytochrome c subunit Vb, putative	-1.280	0.010648	9	59.7	2G03010
NADH-ubiquinone oxidoreductase, subunit G, putative	-1.274	0.035882	33	58.4	5G04370
Fumarylacetoacetase	-1.250	0.007467	23	52	2G04230
N-terminal acetyltransferase catalytic subunit (NAT1), putative	-1.246	0.020861	12	24.3	4G11910
Asparagine synthetase Asn2, putative	-1.218	0.01682	14	23.2	4G06900
Psi-producing oxygenase A (Fatty acid oxygenase PpoA)	-1.212	0.010056	70	61.7	4G10770
Leucine aminopeptidase 2	-1.205	0.03298	29	49.5	2G07520

SsDNA binding protein, putative	-1.181	0.038239	15	72.5	5G07890
Enolase EnoA (allergen Aspf22)	-1.180	0.00727	48	90.2	6G06770
Glutathione S-transferase PsoE	-1.169	0.020172	15	51.1	8G00580
NADH dehydrogenase [ubiquinone] flavoprotein 1, mitochondrial	-1.149	0.00254	20	58.7	4G11050
40S ribosomal protein S19	-1.144	0.043593	10	33.9	1G05340
ATP synthase subunit alpha	-1.137	0.000697	63	77.5	8G05320
Multifunctional fusion protein	-1.132	0.006123	37	58.5	6G08750
Ubiquitin-like modifier SUMO, putative	-1.132	0.045017	8	92.5	1G10850
DUF1989 domain-containing protein	-1.104	0.034703	16	54.6	2G13580
Anthranilate synthase component I, putative	-1.102	0.048457	16	46.4	6G12580
Thioredoxin	-1.087	0.027375	10	72.5	3G14970
Succinate dehydrogenase [ubiquinone] flavoprotein subunit, mitochondrial	-1.082	0.006043	44	68.8	3G07810
Cystathionine gamma-synthase	-1.071	0.005156	5	30.1	7G01590
Allergen Aspf15	-1.042	0.02135	4	32.2	2G12630
Zn-dependent hydrolases of the beta-lactamase fold, putative	Unique to Zn Absence	n/a	7	42.3	1G01460
Glycerol-3-phosphate dehydrogenase [NAD(+)]	Unique to Zn Absence	n/a	11	36.6	1G02150
Proline utilization protein PrnX-like, putative	Unique to Zn Absence	n/a	2	12.5	1G04440
Protein mitochondrial targeting protein (Mas1), putative	Unique to Zn Absence	n/a	5	24.2	1G05040
Protein ignalling 2a 65kd regulatory subunit	Unique to Zn Absence	n/a	7	22.4	1G05610
40S ribosomal protein S23	Unique to Zn Absence	n/a	11	54.5	1G09440
Protein transport protein (LST8), putative	Unique to Zn Absence	n/a	4	22.5	1G09560
Ribosomal protein	Unique to Zn Absence	n/a	3	18.4	1G11710
Oxidoreductase CipA-like, putative	Unique to Zn Absence	n/a	17	68.8	1G12460
Glucoamylase (1,4-alpha-D-glucan glucohydrolase)	Unique to Zn Absence	n/a	10	33.1	2G00690
Fructosyl amine:oxygen oxidoreductase	Unique to Zn Absence	n/a	24	57.3	2G02030
UDP-glucose:glycoprotein glucosyltransferase, putative	Unique to Zn Absence	n/a	6	8.7	2G02360
Alternative oxidase	Unique to Zn Absence	n/a	6	15.3	2G05060
60S ribosomal protein L18	Unique to Zn Absence	n/a	5	31	2G07380
Phenylacetyl-CoA ligase, putative	Unique to Zn Absence	n/a	4	12.1	2G10160

Eukaryotic translation initiation factor 3 subunit L (Eif3l)	Unique to Zn Absence	n/a	3	12.4	2G10380
40S ribosomal protein Rps16, putative	Unique to Zn Absence	n/a	7	46.2	2G10500
Alcohol dehydrogenase, putative	Unique to Zn Absence	n/a	2	4.6	2G10960
BAR domain protein	Unique to Zn Absence	n/a	9	57.7	2G11475
Vesicular-fusion protein sec17	Unique to Zn Absence	n/a	6	36.6	2G12870
Oxidoreductase, putative	Unique to Zn Absence	n/a	5	18	2G14810
DUF323 domain protein	Unique to Zn Absence	n/a	5	10.3	2G15650
N-acetyltransferase domain-containing protein	Unique to Zn Absence	n/a	29	76.9	2G15900
NAD dependent epimerase/dehydratase family protein	Unique to Zn Absence	n/a	11	56.8	3G00330
Mitochondrial enoyl reductase, putative	Unique to Zn Absence	n/a	16	43.4	3G03330
ATP-dependent RNA helicase Dhh1	Unique to Zn Absence	n/a	6	18.9	3G05430
Proteasome regulatory particle subunit (RpnC), putative	Unique to Zn Absence	n/a	3	6.1	3G06110
60S ribosomal protein L21, putative	Unique to Zn Absence	n/a	7	31.6	3G06960
60S ribosomal protein L35Ae	Unique to Zn Absence	n/a	4	31.2	3G08460
Regulatory protein SUAPRGA1	Unique to Zn Absence	n/a	9	59	3G09030
T-complex protein 1, zeta subunit, putative	Unique to Zn Absence	n/a	9	23.9	3G09590
Nonribosomal peptide synthetase 5 NRPS5	Unique to Zn Absence	n/a	10	9.3	3G12920
TAM domain methyltransferase, putative	Unique to Zn Absence	n/a	2	11	3G14920
ThiJ/PfpI family protein	Unique to Zn Absence	n/a	11	44.8	4G01400
Glutathione S-transferase family protein	Unique to Zn Absence	n/a	13	54.6	4G01440
T-complex protein 1, theta subunit, putative	Unique to Zn Absence	n/a	11	29.3	4G09740
Signal recognition particle subunit SRP72	Unique to Zn Absence	n/a	3	5.3	4G10180
RuvB-like helicase 1 Rvb1	Unique to Zn Absence	n/a	6	24.9	4G10730
Carbonic anhydrase	Unique to Zn Absence	n/a	8	25.4	4G11250
Glyceraldehyde-3-phosphate dehydrogenase	Unique to Zn Absence	n/a	41	89.8	5G01030
Amine oxidase	Unique to Zn Absence	n/a	8	18.8	5G01470
WW domain protein	Unique to Zn Absence	n/a	3	21.7	5G03750
Proteasome regulatory particle subunit Rpt2, putative	Unique to Zn Absence	n/a	12	35.4	5G07050
Amidase, putative	Unique to Zn Absence	n/a	18	46.7	5G09140
Oxidoreductase, short chain dehydrogenase/reductase family	Unique to Zn Absence	n/a	9	49	5G14000

Aromatic hydroxylase FmpF	Unique to Zn Absence	n/a	25	53	6G03490
Cell wall integrity ignalling protein Lsp1/Pil1, putative	Unique to Zn Absence	n/a	9	33.9	6G07520
General amidase GmdA, putative	Unique to Zn Absence	n/a	15	35.2	6G08000
Fructosyl amine: oxygen oxidoreductase	Unique to Zn Absence	n/a	21	50	6G10040
Filament-forming protein (Tpr/p270), putative	Unique to Zn Absence	n/a	11	7.2	6G13120
Phospholipase	Unique to Zn Absence	n/a	6	14.3	7G05580
NADPH-dependent FMN reductase Lot6, putative	Unique to Zn Absence	n/a	6	55.9	7G06600
Acetate-CoA ligase, putative	Unique to Zn Absence	n/a	11	28.6	8G00500
Multifunctional cytochrome P450 monooxygenase FmaG	Unique to Zn Absence	n/a	6	20	8G00510
Alpha/beta hydrolase PsoB	Unique to Zn Absence	n/a	12	28.3	8G00530
Cystathionine gamma-lyase	Unique to Zn Absence	n/a	8	33.3	8G04340
